# Dynamics of a Fractional-Order Delayed Model of COVID-19 with Vaccination Efficacy

**DOI:** 10.3390/vaccines11040758

**Published:** 2023-03-29

**Authors:** Fathalla A. Rihan, Udhayakumar Kandasamy, Hebatallah J. Alsakaji, Nicola Sottocornola

**Affiliations:** 1 Department of Mathematical Sciences, College of Science, United Arab Emirates University, Al-Ain 15551, United Arab Emirates; udhayakumar_k@uaeu.ac.ae (U.K.); heba.sakaji@uaeu.ac.ae (H.J.A.); 2 College of Natural and Health Sciences, Zayed University, Abu Dhabi P.O. Box 144534, United Arab Emirates; nicola.spinelli@zu.ac.ae

**Keywords:** COVID-19, fractional-order, time-delay, vaccination, bifurcation, stability

## Abstract

In this study, we provide a fractional-order mathematical model that considers the effect of vaccination on COVID-19 spread dynamics. The model accounts for the latent period of intervention strategies by incorporating a time delay τ. A basic reproduction number, $R_{0}$, is determined for the model, and prerequisites for endemic equilibrium are discussed. The model’s endemic equilibrium point also exhibits local asymptotic stability (under certain conditions), and a Hopf bifurcation condition is established. Different scenarios of vaccination efficacy are simulated. As a result of the vaccination efforts, the number of deaths and those affected have decreased. COVID-19 may not be effectively controlled by vaccination alone. To control infections, several non-pharmacological interventions are necessary. Based on numerical simulations and fitting to real observations, the theoretical results are proven to be effective.

## 1. Introduction

COVID-19 has not been properly controlled for over two years, and the number of new infections remains among the highest ever [1]. According to the World Health Organization (WHO), as of 21 June 2022, there were 187,108,697 confirmed cases globally, with 3,841,225 deaths. Global public health and economic problems are at risk due to the COVID-19 outbreak. In the United Kingdom, South Africa, and Brazil, SARS-CoV-2 variants Alpha (VOC 2020/12/01), Beta (501Y.V2), and Gamma (P.1) have been found. In numerous nations, COVID-19 is spreading faster due to its higher transmission rate [2]. The early stages of COVID-19 were fought with non-pharmaceutical intervention tactics, such as contact tracking, social distancing, isolation, treating sick people, and lockdowns. These restrictions, however, disrupt people’s lives and significantly impede economic development. Consequently, when COVID-19 outbreaks slowed, many countries relaxed these efforts to strengthen their economies. The spread of COVID-19 has not been stopped as a result. To prevent COVID-19’s spread and minimize its impacts on the economy, effective vaccines must be invented and utilized. In order to control COVID-19 effectively and reduce its effects on economic development, people are looking forward to developing and using effective vaccines. Vaccination effectively controls epidemic spread. Several vaccines have been approved for use through the unremitting efforts of all parties, bringing hope that the spread of COVID-19 can be completely controlled [3,4,5,6].

Vaccinations have received relatively little attention in the study of COVID-19 spread. In [7], a mathematical model was used to investigate the impact of a hypothetical ineffective vaccination on COVID-19 control in the United States. A SIRV model was proposed in [3] to predict and model the spread of the COVID-19 outbreak in the presence of vaccination. Reference [8] presents a mathematical model that analyzes the effects of medication (vaccination with complete efficacy) and drug-free prevention strategies on the spread of COVID-19. A wide range of literary studies have been published on COVID-19 models, including review papers, special issues, and books [9,10,11]; however, deterministic modeling of COVID-19 and vaccination is very limited [6]. In mathematical epidemic models, time delays are investigated to better understand and characterize the dynamics of infectious diseases; for example, see [12,13,14,15]. Further, time delays may lead to periodic solutions via the Hopf bifurcation; see, e.g., Reference [16] and references therein. COVID-19 spreads primarily during latent and incubation periods, as with other viral diseases.

Mathematical models can be used to predict and simulate the spread of epidemics and provide a theoretical basis for developing epidemic prevention strategies [4,17,18,19,20,21,22,23]. Many mathematical models have been developed to simulate COVID-19 spread, including those in [24,25,26,27,28,29,30]. Infectious disease research utilizes classical mathematical models with integer-order derivatives, see [31,32,33,34]. Fractional derivatives, however, are advantageous in mathematical models due to their non-locality or long-memory properties [35,36]. COVID-19 dynamics in Lagos, Nigeria, for instance, are described in [37]. The use of fractional derivatives and fractional integrals in epidemiological modeling is important because they can be used to describe memory and hereditary characteristics of various materials and processes [38,39,40,41]. When comparing the fractional-order derivative to the integer-order derivative, it is evident from the literature that fractional operators provide more accurate and deeper results while representing real-life scenarios [42,43,44]. The model used in this work is called the SIR model; it divides the population into three groups, namely susceptible (S), infected (I), and recovered (R). The extensions of this basic model have been applied to COVID-19 in recent times. The main difficulty with this type of model during a pandemic is in determining the model’s parameters. A reasonable estimation of constant and time-dependent parameters can be made using the conventional least squares method. Using a fractional-order mathematical model, we investigated the effects of COVID-19 vaccinations on the dynamics of the disease in a population. The proposed model, in this paper, addresses two new issues: (1) the effects of time delay on infection transmission rates; and (2) vaccination rates in the infected population. We illustrate how the fractional derivative order affects the dynamics of state variables by using the fractional-order differential equations numerical solver in MATLAB. As a result of the study, government, and public health authorities may be able to develop new strategic plans for reducing the spread of COVID-19 outbreaks in the future. The suggested model is examined by fitting real observations in the UAE.

This paper has the following structure. Section 2 presents a delayed fractional-order mathematical model for COVID-19 vaccine efficacy. We provide sufficient conditions for the positivity, boundedness, and uniqueness of mathematical model solutions in Section 2.1. Section 3 provides details of the model’s dynamic analysis. Section 4 provides numerical simulations of the fractional-order vaccination model and the impact of changing the fractional derivative order. Conclusions are found in Section 5.

## 2. Preliminaries and Mathematical Formulation of the Model

The fractional derivatives have several definitions. The Caputo-type fractional derivative is the most common and is utilized in real-world applications. The fractional derivative of a function f(t) with the Caputo type is defined as [45]:

$$\begin{array}{c} D^{\alpha }f\left(t\right)=\frac{1}{\Gamma (1-\alpha )}\int_{0}^{t}{\left(t-\tau \right)}^{n-\alpha -1}f^{\left(n\right)}\left(\varphi \right)d\varphi \end{array}$$

where *n* is the first integer greater than α, i.e., n=[α], Γ(·) is the gamma function.

The α−order Riemann–Liouville integral of a function f(t) is expressed as [45]:

$$\begin{array}{c} I^{\alpha }f\left(t\right)=\frac{1}{\Gamma (\alpha )}\int_{0}^{t}{\left(t-\varphi \right)}^{\alpha -1}f\left(\varphi \right)d\varphi . \end{array}$$

The one-parameter and two-parameter forms of the Mittag-Leffler functions are defined as [38]:

$$E_{\alpha }\left(z\right)=\sum_{k=0}^{\infty }\frac{z^{k}}{\Gamma (\alpha k+1)},\;E_{\alpha ,\beta }\left(z\right)=\sum_{k=0}^{\infty }\frac{z^{k}}{\Gamma (\alpha k+\beta )}$$

where z,α,β∈C.

Numerous researchers have examined various types of models in order to understand the dynamics of COVID-19 using case studies of various specific nations. In order to minimize the likelihood of infection in a susceptible population, vaccination is one of the most effective approaches. Despite the lack of COVID-19 vaccination at birth, the inclusion of it in our theoretical study does not affect the conclusions because some analytical/numerical modeling results are independent of the type of vaccination program used [46]. A graphic representation of the impact of the model settings on initial disease transmission is used to reach this conclusion. COVID-19 has reappeared after numerous waves and strains, and vaccines are still being developed with lower age groups in mind. Since vaccination can involve vaccinating individuals from birth, our proposed strategy is proactive. In order to control epidemics, vaccinations are essential. Globally, several COVID-19 vaccines are in use. In this section, we extend the model proposed by Torku et al. [47] on COVID-19 vaccinations to determine if the disease can be contained by solely relying on the vaccine. The proposed model is governed by a simple system of ODEs.

(1) $$\begin{array}{c} \begin{array}{cccc} \frac{dS}{dt}= & -\delta \frac{SI}{N}-V_{ac}\nu S,\;\frac{dI}{dt}= & \delta \frac{SI}{N}-\beta I,\;\frac{dR}{dt}= & \beta I+V_{ac}\nu S. \end{array} \end{array}$$

At any time *t*, S(t) represents susceptible individuals, I(t) represents infected individuals, and R(t) represents recovered individuals. The first equation presents the rate of change of susceptible individuals in the ordinary model (1). δ represents the transmission rate, ν represents the efficacy rate, and $V_{ac}$ represents the vaccination rate. The second equation presents the rate at which infected individuals change, while β represents the rate at which infected individuals recover. Assume that S(0)≥0,I(0)≥0, and R(0)≥0 are the initial conditions for the model discussed above. The population is assumed to be homogeneous and to have equal chances of becoming infected. This study only considers the human-to-human transmission of COVID-19. N(t) represents the human population at time *t* based on the disease status of people. The N(t) population is divided into three subpopulations: S(t) susceptible individuals, I(t) infected individuals, and R(t) recovered individuals. Based on the vaccination regime, N(t)=S(t)+I(t)+R(t) is assumed to remain constant. Since the population is homogeneous, the standard incidence is $\frac{\delta I(t)}{N(t)}$. Parameter values are given in Table 1.

**Figure 1:**
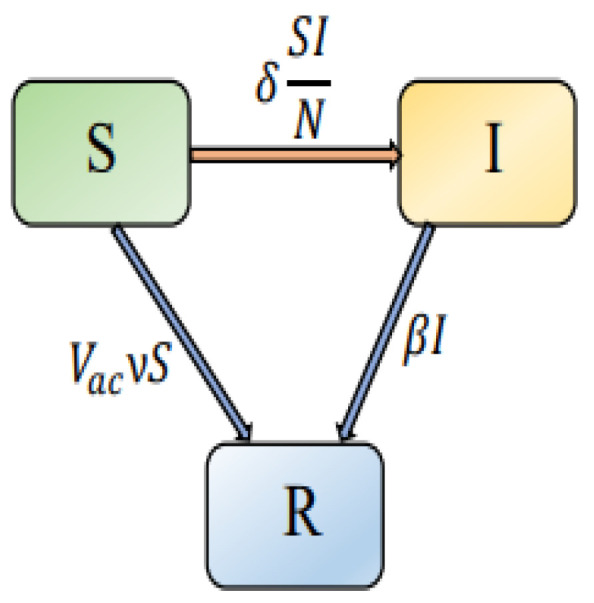
Graphical representation of the interactions between the various elements in the proposed model.

During the course of a disease, time delays occur spontaneously and are significant factors. As a representation of the latent period of the intervention strategies, we include a discrete time-delay τ in system (1). In system (1), human behavior is adapted to intervention tactics. Due to poor knowledge about the disease, people are more likely to be infected when a new infectious disease is discovered. Further, as the number of infected individuals increases and the disease becomes more serious, psychological factors lead people to change their behaviors and implement appropriate measures/interventions to reduce the chances of infection. During infectious disease modeling, delays in intervention processes are significant. In [51], for instance, the length of time for people to react to the reported infection, as well as the delay in reporting, were noted. In addition, the fractional derivative is highly effective in modeling epidemic transition systems since it takes into account the memory effects and the system’s universal features, which are important for deterministic systems. The fractional operator has this memory effect property, making it particularly useful in modeling the COVID-19 model since its future state is dependent on its current state. By substituting the Caputo fractional derivative with the first derivative, we can incorporate past historical or hereditary features into the model. The graphical representation of the interactions between the populations in the proposed model is shown in Figure 1. Thus, the time delay fractional-order differential equations system can be generalized as follows:

(2) $$\begin{array}{c} \begin{array}{cc} D^{\alpha }S\left(t\right)= & -\delta \frac{S(t)I(t-\tau )}{N(t)}-V_{ac}\nu S\left(t\right),\\ D^{\alpha }I\left(t\right)= & \delta \frac{S(t)I(t-\tau )}{N(t)}-\beta I\left(t\right),\\ D^{\alpha }R\left(t\right)= & \beta I\left(t\right)+V_{ac}\nu S\left(t\right). \end{array} \end{array}$$

Although the time-delay system (2) is simple, it provides complex dynamics. For its solution, we should provide initial history conditions: $\theta =(\theta_{1},\theta_{2},\theta_{3})$ defined in terms of space

(3) $$\begin{array}{c} C_{+}=\left\{\theta \in C\left(\left[-\tau ,0\right],R_{0,+}^{3}\right)|\theta_{1}=S\left(r\right),\theta_{2}=I\left(r\right),\theta_{3}=R\left(r\right)\right\}, \end{array}$$

where $S\left(r\right)>0,\;I\left(r\right)>0,\;R\left(r\right)>0,\;r\in C\left[-\tau ,0\right],\;\theta_{1}\left(r\right)\geq 0,\;\theta_{2}\left(r\right)\geq 0,\;\theta_{3}\left(r\right)\geq 0,\;\theta_{1}\left(0\right)>0$, $\theta_{2}\left(0\right)>0,\theta_{3}\left(0\right)>0,C_{+}$ is the Banach space of all continuous functions in the interval [−τ,0] into $R_{0,+}^{3}$, and considering the feasible region of system (2) as

$$\begin{array}{c} R_{0,+}^{3}=\left\{\left(S(t),I(t),R(t)\right)\in R^{3}|S\left(t\right)\geq 0,I\left(t\right)\geq 0,R\left(t\right)\geq 0\right\} \end{array}$$

and $R_{+}^{3}$ is the interior of $R_{0,+}^{3}$

$$\begin{array}{c} R_{+}^{3}=\left\{\left(S(t),I(t),R(t)\right)\in R^{3}|S\left(t\right)>0,I\left(t\right)>0,R\left(t\right)>0\right\}. \end{array}$$

Thus, the region is positively invariant with respect to system (2), which means that all solutions of model (2) are contained within the above region for all time *t*, and those outside are ultimately attracted to it. In this sense, system (2) has been posed appropriately from an epidemiological perspective.

**Remark** **1.** *Is it possible to completely eliminate COVID-19 through vaccination? A fractional-order model with time delay is used here to analyze it. As the infectious disease outbreak spreads, fractional-order models are very useful for evaluating the efficiency of several interventions, such as vaccination and lockdown. In this study, the primary objective is to evaluate the efficacy of COVID-19 infection vaccination strategies using the fractional-order model with the Caputo-type derivative. Memory is accounted for by the fractional order.*

### 2.1. Positivity, Boundedness, and Uniqueness of the Solution

Positivity is necessary for biologically feasible model solutions, whereas boundedness indicates that solutions are finite.

**Lemma** **1.** *(Positivity) If S(0)≥0,I(0)≥0,R(0)≥0, then the solution of the fractional-order model (2) remains non-negative for all positive times t.*

**Proof.**  From model (2), we have

(4) $$\begin{array}{c} D^{\alpha }S\left(t\right)\geq -V_{ac}\nu S\left(t\right). \end{array}$$

Hence, one derives

(5) $$\begin{array}{c} S\left(t\right)\geq S\left(t_{0}\right)E_{\alpha }\left(-V_{ac}\nu {\left(t-t_{0}\right)}^{\alpha }\right). \end{array}$$

Since $S(t_{0})\geq 0$, one obtains S(t)≥0 for any $t>t_{0}.$ Thus, S(t) remains non-negative ∀t>0. In the same manner, I(t)≥0,R(t)≥0 are all non-negative. This completes the proof. □

Our next step is to demonstrate that the model system solutions (2) enter a bounded region.

**Lemma** **2.** *(Boundedness) All of system (2)’s solutions with non-negative initial history conditions are bounded.*

**Proof.**  To show that system (2) is bounded, the population growth can be expressed as

(6) $$\begin{array}{c} D^{\alpha }N\left(t\right)=D^{\alpha }S\left(t\right)+D^{\alpha }I\left(t\right)+D^{\alpha }R\left(t\right). \end{array}$$

It can be seen from (6) that

(7) $$\begin{array}{c} D^{\alpha }N\left(t\right)=-V_{ac}\nu N\left(t\right) \end{array}$$

where N(t)=S(t)+I(t)+R(t). Since the human population N(t) is positive, by solving Equation (7), the total human population satisfies the following equation:

(8) $$\begin{array}{c} N\left(t\right)=N\left(t_{0}\right)E_{\alpha }\left(-V_{ac}\nu t^{\alpha }\right). \end{array}$$

The solution is given by $N\left(t\right)=N\left(t_{0}\right)E_{\alpha ,1}\left(-V_{ac}\nu t^{\alpha }\right),$ where $E_{\alpha ,\eta }$ is the Mittag-Leffler function. The Mittag-Leffler function $E_{\alpha ,\eta }$ is asymptotic in nature; knowing that, the asymptotic behavior of the Mittag-Leffler function is as follows:

(9) $$\begin{array}{c} E_{\alpha ,\eta }\left(u\right)=-\sum_{p=1}^{n}\frac{u^{-p}}{\Gamma (\eta -\alpha p)}+O\left({\left|u\right|}^{-1-n}\right),\mathrm{as}|u|\to \infty ,\frac{\alpha \pi }{2}<\left|\arg\left(u\right)\right|\leq \pi . \end{array}$$

In particular, $N\left(t\right)=N\left(t_{0}\right)\exp\left(-V_{ac}\nu t\right)$ for α=1, i.e., the exponential function. In light of this, all of system (2)’s solutions with non-negative initial history conditions remain bounded. The proof is completed. □

**Lemma** **3.** *(Uniqueness) For every $(S\left(t_{0}\right),I\left(t_{0}\right),R\left(t_{0}\right))\in \Delta ,$ there exists a unique solution W(t)=(S(t),I(t),R(t))∈Δ of system (2), where $\Delta =\left\{\left(S\left(t\right),I\left(t\right),R\left(t\right)\right)\in R^{3}|\max\left\{|S(t)|,\right.\right.$|I(t)|,$\left.\left.|R(t)|\leq U\right\}\right\}.$*

**Proof.**  Based on the Banach space of all continuous and differentiable functions from [0,T]→R, we demonstrate that F(W) is Lipschitz continuous with Lipschitz constants using the fundamental fixed-point theorem. Based on a triangle inequality and the Chebyshev norm, let $W_{1}\left(t\right)$ be the second solution.

Consider the contraction mapping

$$\begin{array}{cc} F(W)= & (F_{1}\left(W\right),F_{2}\left(W\right),F_{3}\left(W\right)),\\ F_{1}\left(W\right)= & -\delta \frac{S(t)I(t-\tau )}{N}-V_{ac}\nu S\left(t\right),\\ F_{2}\left(W\right)= & \delta \frac{S(t)I(t-\tau )}{N}-\beta I\left(t\right),\\ F_{3}\left(W\right)= & \beta I\left(t\right)+V_{ac}\nu S\left(t\right). \end{array}$$

Then, for any vectors $W,W_{1}\in \Delta$,

(10) $$\begin{array}{cc} ∥F\left(W\right)-F\left(W_{1}\right)∥ & =\left|\frac{\delta }{N}\left(S\left(t\right)I\left(t\right)-S_{1}\left(t\right)I_{1}\left(t\right)\right)+V_{ac}\nu \left(S\left(t\right)-S_{1}\left(t\right)\right)\right|\\ & \;\;\;+\left|\frac{\delta }{N}\left(S\left(t\right)I\left(t\right)-S_{1}\left(t\right)I_{1}\left(t\right)\right)+\beta \left(I\left(t\right)-I_{1}\left(t\right)\right)\right|\\ & \;\;\;+\left|V_{ac}\nu \left(S\left(t\right)-S_{1}\left(t\right)\right)+\beta \left(I\left(t\right)-I_{1}\left(t\right)\right)\right|,\\ & \leq 2\left(\frac{\delta }{N}U+V_{ac}\nu \right)∥S\left(t\right)-S_{1}\left(t\right)|+2\left(\frac{\delta }{N}U+2\beta \right)∥I\left(t\right)-I_{1}\left(t\right)|,\\ & \leq G∥W-W_{1}∥ \end{array}$$

where $G=\max\left\{2\left(\frac{\delta }{N}U+V_{ac}\nu \right),2\left(\frac{\delta }{N}U+2\beta \right),0\right\}.$ Thus, with the agreement in (10) and Lemma 5 in [52], F(W) satisfies the Lipschitz condition in its second argument with the Lipschitz constant G, then model (2) has a unique solution W(t). □

### 2.2. Equilibrium Points (Disease-Free and Endemic)

In this subsection, we explore the existence of equilibrium points. According to (2), the stability analysis of model (2) is carried out to determine the disease-free and endemic equilibrium point. Every equation in (2) needs to be equated to zero in order to establish the equilibrium points, or $D^{\alpha }S\left(t\right)=0,D^{\alpha }I\left(t\right)=0,D^{\alpha }R\left(t\right)=0$, achieved as follows:

(11) $$\begin{array}{c} \begin{array}{c} l-\delta \frac{SI(t-\tau )}{N}-V_{ac}\nu S=0,\;\delta \frac{SI(t-\tau )}{N}-\beta I=0,\;\beta I+V_{ac}\nu S=0. \end{array} \end{array}$$

Then the equilibrium point of S(t),I(t),R(t) is determined. The equilibrium where the number of infected individuals is zero is the so-called disease-free equilibrium. When COVID-19 is not spreading, the conditions that define the disease-free equilibrium are met, which means I(t)=0. Using (11), we obtain S(t)=0. Therefore, the disease-free equilibrium points for the COVID-19 vaccination model are: $E_{0}=\left(S_{0},I_{0}\right)=\left(0,0\right)$. By taking into account the scenario in which I(t) is positive, we can identify the endemic equilibria of the model. Endemic equilibrium points are used to predict whether a disease will continue to spread because populations S(t)≠0 and I(t)≠0 under endemic conditions when the disease is spreading. Solving for S(t) and I(t) in Equation (11), the endemic equilibrium points for the vaccination model were determined:

(12) $$\begin{array}{cc} S_{★}= & \frac{\beta N}{\delta },I_{★}=-\frac{V_{ac}\nu N}{\delta }. \end{array}$$

Then, the COVID-19 vaccination model equilibrium points of the endemic are:

(13) $$\begin{array}{c} E_{★}=\left(S_{★},I_{★}\right)=\left(\frac{\beta N}{\delta },-\frac{V_{ac}\nu N}{\delta }\right). \end{array}$$

### 2.3. Basic Reproduction Number $R_{0}$

The basic reproduction number, $R_{0}$, is an epidemiologically significant threshold value that predicts the probability that infectious disease will spread throughout a population. The matrix generation method is used to determine the basic reproduction number $R_{0}$. Using Equation (2), we determine $R_{0}.$ The compartments of model (2) consist of (S(t),I(t),R(t)) classes if we take $X\left(t\right)={\left(S\left(t\right),I\left(t\right),R\left(t\right)\right)}^{T}$; we now want to write the infection subsystem in the following form:

(14) $$\begin{array}{c} D^{\alpha }X\left(t\right)=Z\left(S\left(t\right)\right)X\left(t\right) \end{array}$$

which is equivalent to

(15) $$\begin{array}{c} \left[\begin{array}{c} D^{\alpha }S\left(t\right)\\ D^{\alpha }I\left(t\right)\\ D^{\alpha }R\left(t\right) \end{array}\right]=\left[\begin{array}{ccc} -V_{ac}\nu & -\delta \frac{S(t)}{N(t)} & 0\\ 0 & \delta \frac{S(t)}{N(t)}-\beta & 0\\ V_{ac}\nu & \beta & 0 \end{array}\right]\left[\begin{array}{c} S(t)\\ I(t)\\ R(t) \end{array}\right]. \end{array}$$

Let P(t)=I(t), system (15) can be rewritten in the following form:

(16) $$\begin{array}{c} \left\{\begin{array}{cc} D^{\alpha }P\left(t\right)= & FP(t)+lQ(t),\\ Y(t)= & dP(t),\\ Q(t)= & S(t)Y(t) \end{array}\right. \end{array}$$

where F,l, and *d* are defined as F=−β,l=1,d=δ.

The following differential equations are satisfied by the remaining variables:

(17) $$\begin{array}{c} \left\{\begin{array}{cc} D^{\alpha }S\left(t\right)= & -Q\left(t\right)-V_{ac}\nu S\left(t\right),\\ D^{\alpha }R\left(t\right)= & \gamma P\left(t\right)+V_{ac}\nu S\left(t\right). \end{array}\right. \end{array}$$

The expected number of secondary cases produced by a single infected person over the course of his/her infectiousness in a population that is totally susceptible is known as the basic reproduction number $R_{0}$. Then from (17), using the approach of matrices generation method, we obtained the basic reproduction number $R_{0}$ of (2) as

(18) $$\begin{array}{c} R_{0}=-dF^{-1}l=\frac{\delta }{\beta }. \end{array}$$

$R_{0}$ is denoted as the basic reproduction number in the without-vaccination cases. In a vaccination scenario, the current reproduction number $R_{t}$ is defined as the reproduction number with respect to time. It is calculable as

(19) $$\begin{array}{c} R_{t}=\frac{\delta_{t}}{\beta_{t}}. \end{array}$$

$R_{t}$ depends on a time-varying recovery rate $\beta_{t}$ and transmission rate $\delta_{t}$. The effective reproduction number $R_{e}$ is defined as $R_{e}=R_{0}\frac{S(t)}{N(t)},$ where S(t) is the number of susceptible people, N(t) is the population density of a certain location, and $R_{0}$ is the basic reproduction number at a given point in time.

## 3. Stability and Hopf Bifurcation Analyses

This section focuses on the local stability and bifurcation analysis of model (20). For the local asymptotic stability analysis, let us reduce system (2) by discarding the last equation as R(t) does not appear in the first two equations of model (2). If we study the qualitative or dynamic behaviors of S(t),I(t), then the dynamic behaviors of R(t) are also obtained from the dynamic behaviors of S(t)I(t). Here is the simplified fractional system:

(20) $$\begin{array}{c} \begin{array}{cc} D^{\alpha }S\left(t\right)= & -\delta \frac{S(t)I(t-\tau )}{N}-V_{ac}\nu S\left(t\right),\\ D^{\alpha }I\left(t\right)= & \delta \frac{S(t)I(t-\tau )}{N}-\beta I\left(t\right). \end{array} \end{array}$$

The equilibrium points of model (20) are defined in Section 2.2. The method of linearization entails taking a nonlinear function’s gradient with regard to each variable and converting it into a linear representation at that point. It is necessary for some analyses, including stability analysis, Laplace transform solutions, and putting the model into a linear state-space form. Consider the differential model (20). The right-hand side of the model can be linearized at any steady-state $E_{★}\left(S_{★},I_{★}\right)$ using a Taylor series expansion, which involves only the first two terms.

(21) $$\begin{array}{c} \begin{array}{cc} D^{\alpha }S\left(t\right)= & -\left(\frac{\delta I_{★}}{N}+V_{ac}\nu \right)S\left(t\right)-\left(\frac{\delta S_{★}}{N}\right)I\left(t-\tau \right),\\ D^{\alpha }I\left(t\right)= & \left(\frac{\delta I_{★}}{N}\right)S\left(t\right)+\left(\frac{\delta S_{★}}{N}\right)I\left(t-\tau \right)-\beta I\left(t\right). \end{array} \end{array}$$

Next, we take the Laplace transform on both sides of (21) to obtain

(22) $$\begin{array}{cc} \left(\lambda^{\alpha }+\frac{\delta I^{★}}{N}+V_{ac}\nu \right)S\left(\lambda \right)+\left(\frac{\delta S_{★}}{N}\right)I\left(\lambda \right)= & \lambda^{\alpha -1}\theta \left(0\right)+\left(\frac{\delta S_{★}}{N}\right)\left(-\int_{-\tau }^{0}e^{-\lambda t}\vartheta \left(t\right)dt\right),\\ -\left(\frac{\delta I_{★}}{N}\right)S\left(\lambda \right)+\left(\lambda^{\alpha }+\beta -\frac{\delta S_{★}}{N}\right)I\left(\lambda \right)= & \lambda^{\alpha -1}\vartheta \left(0\right)+\left(\frac{\delta S_{★}}{N}\right)\left(\int_{-\tau }^{0}e^{-st}\vartheta \left(t\right)dt\right) \end{array}$$

where the Laplace transforms of S(t) and I(t) are S(λ) and I(t), respectively. Then, Equation (22) can be written as

(23) $$\begin{array}{cc} \Lambda \left(\lambda \right)\cdot \left(\begin{array}{c} S(\lambda )\\ I(\lambda ) \end{array}\right)= & \left(\begin{array}{c} \lambda^{\alpha -1}\theta \left(0\right)-\left(\frac{\delta S_{★}}{N}\right)\left(\int_{-\tau }^{0}e^{-\lambda t}\vartheta \left(t\right)dt\right)\\ \lambda^{\alpha -1}\vartheta \left(0\right)+\left(\frac{\delta S_{★}}{N}\right)\left(\int_{-\tau }^{0}e^{-st}\vartheta \left(t\right)dt\right) \end{array}\right) \end{array}$$

where

(24) $$\begin{array}{c} \Lambda \left(\lambda \right)=\left(\begin{array}{cc} \lambda^{\alpha }+\frac{\delta I_{★}}{N}+V_{ac}\nu & \frac{\delta S_{★}}{N}e^{-\lambda \tau }\\ -\left(\frac{\delta I_{★}}{N}\right) & \lambda^{\alpha }+\beta -\frac{\delta S_{★}}{N}e^{-\lambda \tau } \end{array}\right) \end{array}$$

Λ(λ) is the characteristic matrix of system (20) at $E_{★}\left(S_{★},I_{★}\right).$

The characteristic equation of (20) at the disease-free equilibrium $E_{0}\left(0,0\right)$ is represented by

(25) $$\begin{array}{c} \lambda^{2\alpha }+\lambda^{\alpha }\left(V_{ac}\nu +\frac{\delta }{R_{0}}\right)+\frac{V_{ac}\delta \nu }{R_{0}}=0. \end{array}$$

Now, as observed is the fact that if α=1, then the above characteristic equation becomes

(26) $$\begin{array}{c} \lambda^{2}+\lambda \left(V_{ac}\nu +\frac{\delta }{R_{0}}\right)+\frac{V_{ac}\delta \nu }{R_{0}}=0 \end{array}$$

which has two roots

(27) $$\begin{array}{cc} \lambda_{1,2}= & \frac{1}{2R_{0}}\left[-\left(V_{ac}\nu R_{0}+\delta \right)\pm \sqrt{{\left(V_{ac}\nu R_{0}\right)}^{2}-4V_{ac}\nu \delta R_{0}}\right]. \end{array}$$

When all coefficients of the characteristic Equation (26) are positive, both the roots in (27) will be negative. Therefore, the disease-free equilibrium $E_{0}\left(0,0\right)$ is locally asymptotically stable. In case 0<α<1, the characteristic Equation (25) has the following roots

(28) $$\begin{array}{cc} \lambda^{\left(1\right)}= & {\left(2R_{0}\right)}^{-\alpha }{\left(\sqrt{V_{ac}\nu R_{0}}\left(V_{ac}\nu R_{0}-4\delta \right)-\delta -V_{ac}\nu R_{0}\right)}^{\frac{1}{\alpha }},\\ \lambda^{\left(2\right)}= & -{\left(2R_{0}\right)}^{-\alpha }{\left(\sqrt{V_{ac}\nu R_{0}}\left(V_{ac}\nu R_{0}-4\delta \right)+V_{ac}\nu R_{0}+\delta \right)}^{\frac{1}{\alpha }}, \end{array}$$

and the equilibrium is locally asymptotically stable. The stability conditions of the infection-free steady-state are presented in the following theorem.

**Theorem** **1.** *The characteristic equation at the disease-free equilibrium has two negative roots, when 0<α<1, $\left(V_{ac}\nu +\frac{\delta }{R_{0}}\right)>0,$ and $\frac{V_{ac}\delta \nu }{R_{0}}>0$. Then the disease-free equilibrium $E_{0}\left(0,0\right)$ is locally asymptotically stable.*

**Remark** **2.** *Eventually, the disease will disappear if $R_{0}$ is less than 1. If $R_{0}$ is greater than 1, severe effects will result. When $R_{0}=1$, the disease is spreading steadily and persistently. Increasing (decreasing) the parameter δ (β) leads to an increase in the basic reproduction number $R_{0}$ from (18). A small change in any of these factors can result in a large variation in the reproduction number $R_{0}$.*

**Remark** **3.** *All of the roots of Equation (25) have negative real parts due to $R_{0}<1,V_{ac}\nu >0$. The disease-free equilibrium $E_{0}\left(0,0\right)$ is locally asymptotically stable when α=1. If $R_{0}>1,V_{ac}\nu <0$, Equation (25) has a positive root. As a result, the disease-free equilibrium $E_{0}\left(0,0\right)$ is unstable.*

Now we will look at the local stability of an endemic equilibrium $E_{★}=\left(S_{★},I_{★}\right)$. Because $S_{★}=\frac{\beta N}{\delta }=\frac{N}{R_{0}}$ and $I_{★}=-\frac{V_{ac}\nu N}{\delta }=-\frac{V_{ac}\nu N}{R_{0}\beta }$, then the following characteristic equation is obtained at endemic equilibrium $E_{★}=\left(S_{★},I_{★}\right)$

(29) $$\begin{array}{c} {\left(\lambda^{\alpha }\right)}^{2}+\lambda^{\alpha }\left(2\beta -\beta e^{-\lambda \tau }\right)-e^{-\lambda \tau }V_{ac}\nu \beta =0 \end{array}$$

or

(30) $$\begin{array}{c} \left(\lambda^{2\alpha }+2\beta \lambda^{\alpha }\right)-e^{-\lambda \tau }\left(\lambda^{\alpha }\beta +q\right)=0. \end{array}$$

Let us proceed with $C_{1}=2\beta -\beta e^{-\lambda \tau },C_{2}=-e^{-\lambda \tau }V_{ac}\nu \beta$, then (29) takes the following form

(31) $$\begin{array}{c} {\left(\lambda^{\alpha }\right)}^{2}+\lambda^{\alpha }C_{1}+C_{2}=0. \end{array}$$

When τ=0, it is important to note that the Routh–Hurwitz criterion provides both sufficient and necessary conditions for roots of (31) to have negative real parts and $C_{1}>0$, $C_{2}>0$ are the conditions. Therefore, if $C_{1}>0,C_{2}>0,$, the equilibrium point $E_{★}=\left(S_{★},I_{★}\right)$ is locally asymptotically stable.

It will then be verified that det(Λ(λ)) does not have any pure imaginary roots for any τ>0. The fact is testified by contradiction. Assume that there exists a pure imaginary root $\lambda =i\omega =\omega \left(\cos\frac{\pi }{2}+i\sin\frac{\pi }{2}\right)$ for (30), where ω is a real positive number. When τ≠0, we substitute the pure imaginary root λ=iω in the Equation (30), obtaining

(32) $$\begin{array}{cc} \; & \left({\left(\omega \right)}^{2\alpha }\cos\frac{2\alpha \pi }{2}+2\omega^{\alpha }\beta \cos\frac{\alpha \pi }{2}\right)+i\left({\left(\omega \right)}^{2\alpha }\sin\frac{2\alpha \pi }{2}+2\omega^{\alpha }\beta \sin\frac{\alpha \pi }{2}\right)\\ & -\left(\cos\omega \tau -i\sin\omega \tau \right)\left(\left(\beta \omega^{\alpha }\cos\frac{\alpha \pi }{2}+q\right)+i\left(\beta \omega^{\alpha }\sin\frac{\alpha \pi }{2}\right)\right)=0. \end{array}$$

Separating the real and imaginary components of (32) results in

(33) $$\begin{array}{c} \left(\beta \omega^{\alpha }\cos\frac{\alpha \pi }{2}+q\right)\cos\omega \tau +\left(\beta \omega^{\alpha }\sin\frac{\alpha \pi }{2}\right)\sin\omega \tau =\left({\left(\omega \right)}^{2\alpha }\cos\frac{2\alpha \pi }{2}+2\omega^{\alpha }\beta \cos\frac{\alpha \pi }{2}\right), \end{array}$$

(34) $$\begin{array}{c} \left(\beta \omega^{\alpha }\sin\frac{\alpha \pi }{2}\right)\cos\omega \tau -\left(\beta \omega^{\alpha }\cos\frac{\alpha \pi }{2}+q\right)\sin\omega \tau =\left({\left(\omega \right)}^{2\alpha }\sin\frac{2\alpha \pi }{2}+2\omega^{\alpha }\beta \sin\frac{\alpha \pi }{2}\right). \end{array}$$

Using Cramer’s rule to solve (33) and (34), one obtains

(35) $$\begin{array}{cc} \cos\omega \tau = & \frac{\beta \cos\left(\frac{3\alpha \pi }{2}\right)\omega^{3\alpha }+\left(q+2\beta^{2}\right)\cos\alpha \pi \omega^{2\alpha }+2q\beta \cos\left(\frac{\alpha \pi }{2}\right)\omega^{\alpha }}{\beta^{2}\cos\alpha \pi \omega^{2\alpha }+2q\beta \cos\left(\frac{\alpha \pi }{2}\right)\omega^{\alpha }+q^{2}}, \end{array}$$

(36) $$\begin{array}{cc} \sin\omega \tau = & \frac{\beta \sin\left(\frac{\alpha \pi }{2}\right)\omega^{3\alpha }+q\sin\alpha \pi \omega^{2\alpha }+2q\beta \sin\left(\frac{\alpha \pi }{2}\right)\omega^{\alpha }}{\beta^{2}\cos\alpha \pi \omega^{2\alpha }+2q\beta \cos\left(\frac{\alpha \pi }{2}\right)\omega^{\alpha }+q^{2}}. \end{array}$$

Therefore,

(37) $$\begin{array}{c} \cos2\alpha \pi \omega^{4\alpha }+4\beta \cos\frac{3\alpha \pi }{2}\omega^{3\alpha }+\left(4\beta^{2}\cos\alpha \pi -\beta^{2}\right)\omega^{2\alpha }-2q\beta \cos\frac{\alpha \pi }{2}\omega^{\alpha }+a_{4}=0. \end{array}$$

Let

(38) $$\begin{array}{c} f\left(\omega \right)=\cos2\alpha \pi \omega^{4\alpha }+a_{1}\omega^{3\alpha }+a_{2}\omega^{2\alpha }+a_{3}\omega^{\alpha }+a_{4} \end{array}$$

and

(39) $$\begin{array}{c} g\left(\delta \right)=\cos2\alpha \pi \delta^{4}+a_{1}\delta^{3}+a_{2}\delta^{2}+a_{3}\delta +a_{4} \end{array}$$

where $a_{1}=4\beta \cos\frac{3\alpha \pi }{2},a_{2}=\left(4\beta^{2}\cos\alpha \pi -\beta^{2}\right),a_{3}=-2q\beta \cos\frac{\alpha \pi }{2},a_{4}={\left(V_{ac}\nu \beta \right)}^{2}.$

If the condition

$$\begin{array}{c} \left(H1\right)\;\;\;\;\;a_{4}<0 \end{array}$$

holds, according to $\frac{df(\omega )}{d\omega }$ for all ω>0. It is evident that $\lim_{\omega \to \infty }f\left(\omega \right)=\infty .$ Then if $a_{4}<0$, then (37) has at least one positive real root. Therefore, (32) has at least one pair of purely imaginary roots.

If τ=0. Then (32) becomes

(40) $$\begin{array}{c} \chi^{2}+\chi \frac{\beta (\cos\frac{\alpha \pi }{2}+3\sin\frac{\alpha \pi }{2})}{\cos\frac{2\alpha \pi }{2}+\sin\frac{2\alpha \pi }{2}}+\frac{q}{\frac{\cos2\alpha \pi }{2}+\sin\frac{2\alpha \pi }{2}}=0 \end{array}$$

where $q=V_{ac}\nu \beta .$ If the condition

$$\begin{array}{c} \left(H2\right)\;\frac{\beta (\cos\frac{\alpha \pi }{2}+3\sin\frac{\alpha \pi }{2})}{\cos\frac{2\alpha \pi }{2}+\sin\frac{2\alpha \pi }{2}}>0,\frac{q}{\frac{\cos2\alpha \pi }{2}+\sin\frac{2\alpha \pi }{2}}>0 \end{array}$$

holds, then all the roots $\chi_{1},\chi_{2}$ of (40) satisfy $|\arg\left(\chi_{i}\right)|>\frac{\alpha \pi }{2},i=1,2.$ Therefore, we can deduce that the endemic equilibrium point $E_{★}=\left(S_{★},I_{★}\right)$ of (20) with τ=0 is locally asymptotically stable.

**Lemma** **4.** *The characteristic Equation (37) of model (20) has no pure imaginary roots if $a_{1}>0,a_{2}>0,a_{3}>0$ and $a_{4}>0$. If $a_{4}<0$, then the characteristic matrix of model (20) has at least a pair of purely imaginary roots. If $a_{4}>0$ and there exists a positive constant $\delta_{0}$ such that the derivative $g^{\prime }\left(\delta_{0}\right)<0$, then the characteristic Equation (37) has at least two pairs of purely imaginary roots.*

**Proof.**  In light of $a_{1},a_{2},a_{3}>0$ and $a_{4}>0$, then $\frac{df(\omega )}{d\omega }>0$ for all ω>0 and $f\left(0\right)=a_{4}>0.$ Then we already know that (37) has no positive real root. Therefore, (32) has no pure imaginary root. In light of the condition (H2), λ=0 is not the solution of the characteristic Equation (32). Therefore, the characteristic Equation (32) does not have any root with a zero real part. Evidently, $f\left(0\right)=a_{4}<0,$ and $\lim_{\omega \to \infty }f\left(\omega \right)=+\infty$. Then (37) has at least one positive real root. Therefore, Equation (32) has at least one pair of purely imaginary roots. Moreover, we know that $g\left(0\right)=a_{4}>0,$ and some positive constant $\varrho_{0}$, then the derivative $g^{\prime }\left(\varrho_{0}\right)<0$ and $\lim_{\delta \to \infty }\frac{g(\delta )}{d\delta }=+\infty$, then there exists two constants, $\delta_{01}\in \left(0,\delta_{0}\right)$ and $\delta_{02}\in \left(\delta_{0},+\infty \right)$, such that $g\left(\delta_{01}\right)=g\left(\delta_{02}\right)=0$, which implies that (37) has at least two positive real roots. Then the characteristic Equation (32) has at least two pairs of purely imaginary roots. This completes the proof of Lemma 4. □

We suppose that (37) has a positive real root ω. By (35), we obtain

(41) $$\begin{array}{c} \tau_{h}^{r}=\frac{1}{\omega_{h}}\left[arccos\left(\frac{\beta \cos\left(\frac{3\alpha \pi }{2}\right)\omega^{3\alpha }+\left(q+2\beta^{2}\right)\cos\alpha \pi \omega^{2\alpha }+2q\beta \cos\left(\frac{\alpha \pi }{2}\right)\omega^{\alpha }}{\beta^{2}\cos\alpha \pi \omega^{2\alpha }+2q\beta \cos\left(\frac{\alpha \pi }{2}\right)\omega^{\alpha }+q^{2}}\right)+2r\pi \right] \end{array}$$

where h=1,2,3,4;r=0,1,2,…. Then ±iω is the pair of purely imaginary roots of (32) when $\tau =\tau_{h}^{r}$, denoting $\tau_{0}=\tau_{h_{0}}^{0}=\min\left\{\tau_{h}^{0}|h=1,2,3,4\right\}$ and $\omega_{0}=\omega_{h_{0}}.$

**Lemma** **5.** *If we write (38) as $f\left(\omega \right)=\omega^{4\alpha }+{\bar{a}}_{1}\omega^{3\alpha }+{\bar{a}}_{2}\omega^{2\alpha }+{\bar{a}}_{3}\omega^{\alpha }+{\bar{a}}_{4},$ where* ${\bar{a}}_{p}=a_{p}/\cos2\alpha \pi$ *, p=1,2,3,4, if (H2) holds and ${\bar{a}}_{1}>0,{\bar{a}}_{2}>0,{\bar{a}}_{3}>0,{\bar{a}}_{4}>0$, then (32) has no root with zero real parts when τ≥0. If ${\bar{a}}_{4}<0$ and ${\bar{a}}_{1},{\bar{a}}_{2},{\bar{a}}_{3}>0$, then (32) has a pair of purely imaginary roots $\pm i\omega_{0}$ when $\tau =\tau_{h}^{r}$, where $\omega_{0}$ is the unique positive zero solution of the function f(ω).*

We give the following assumption

$$\begin{array}{c} \left(H3\right)\;A_{1}B_{1}+A_{2}B_{2}>0 \end{array}$$

where

$$\begin{array}{cc} A_{1}= & \alpha \beta \omega_{0}^{\alpha -1}\cos\omega_{0}\tau_{0}\cos\frac{(\alpha -1)\pi }{2}+\alpha \beta \omega_{0}^{\alpha -1}\sin\omega_{0}\tau_{0}\sin\frac{(\alpha -1)\pi }{2}\\ & -2\alpha \omega_{0}^{2\alpha -1}\cos\frac{(2\alpha -1)\pi }{2}-2\alpha \beta \omega_{0}^{\alpha -1}\cos\frac{(\alpha -1)\pi }{2},\\ B_{1}= & \alpha \beta \omega_{0}^{\alpha -1}\cos\omega_{0}\tau_{0}\sin\frac{(\alpha -1)\pi }{2}-\alpha \beta \omega_{0}^{\alpha -1}\sin\omega_{0}\tau_{0}\cos\frac{(\alpha -1)\pi }{2}\\ & -2\alpha \omega_{0}^{2\alpha -1}\sin\frac{(2\alpha -1)\pi }{2}-2\alpha \beta \omega_{0}^{\alpha -1}\sin\frac{(\alpha -1)\pi }{2},\\ A_{2}= & \beta \omega_{0}^{\alpha +1}\sin\omega_{0}\tau_{0}\cos\frac{\alpha \pi }{2}-\beta \omega_{0}^{\alpha +1}\cos\omega_{0}\tau_{0}\sin\frac{\alpha \pi }{2}+q\omega_{0}\sin\omega_{0}\tau_{0},\\ B_{2}= & \beta \omega_{0}^{\alpha +1}\cos\omega_{0}\tau_{0}\cos\frac{\alpha \pi }{2}+\beta \omega_{0}^{\alpha +1}\sin\omega_{0}\tau_{0}\sin\frac{\alpha \pi }{2}+q\omega_{0}\cos\omega_{0}\tau_{0}. \end{array}$$

**Lemma** **6.** *If λ(τ)=α(τ)+iω(τ) is the root of (32) near $\tau =\tau_{h_{0}}^{0}$, satisfying $\alpha (\tau_{h_{0}}^{0})=0$ and $\omega \left(\tau_{h_{0}}^{0}\right)=\omega_{h_{0}}$, then the following criterion holds*

$$\begin{array}{c} Re\left(\frac{d\lambda }{d\tau }\right){|}_{\tau =\tau_{0},\omega =\omega_{0}}>0. \end{array}$$

**Proof.**  By (32), one has

(42) $$\begin{array}{c} {\left(\frac{d\lambda }{d\tau }\right)}^{-1}=\frac{\alpha \beta \lambda^{\alpha -1}e^{-\lambda \tau }-\left(2\alpha \lambda^{2\alpha -1}+2\alpha \beta \lambda^{\alpha -1}\right)}{\lambda \left(\lambda^{\alpha }\beta +q\right)e^{-\lambda \tau }}-\frac{\tau }{\lambda }. \end{array}$$

Then

(43) $$\begin{array}{c} Re\left[{\left(\frac{d\lambda }{d\tau }\right)}^{-1}\right]=Re\left[\frac{\alpha \beta \lambda^{\alpha -1}e^{-\lambda \tau }-\left(2\alpha \lambda^{2\alpha -1}+2\alpha \beta \lambda^{\alpha -1}\right)}{\lambda \left(\lambda^{\alpha }\beta +q\right)e^{-\lambda \tau }}\right]. \end{array}$$

Thus,

(44) $$\begin{array}{cc} Re\left[{\left(\frac{d\lambda }{d\tau }\right)}^{-1}\right]{|}_{\tau =\tau_{0},\omega =\omega_{0}}= & Re{\left[\frac{\alpha \beta \lambda^{\alpha -1}e^{-\lambda \tau }-\left(2\alpha \lambda^{2\alpha -1}+2\alpha \beta \lambda^{\alpha -1}\right)}{\lambda \left(\lambda^{\alpha }\beta +q\right)e^{-\lambda \tau }}\right]}_{\tau =\tau_{0},\omega =\omega_{0}},\\ = & \frac{A_{1}B_{1}+A_{2}B_{2}}{B_{1}^{2}+B_{2}^{2}}. \end{array}$$

By condition (H3), one has

$$\begin{array}{c} Re\left(\frac{d\lambda }{d\tau }\right){|}_{\tau =\tau_{0},\omega =\omega_{0}}>0. \end{array}$$

The proof is now completed. □

**Theorem** **2.** *Based on the discussion for system (2), if τ>0 and (H1)−(H3) hold, then the endemic equilibrium $E_{★}=\left(S_{★},I_{★}\right)$ is locally asymptotically stable when $\tau \in [0,\tau_{0})$ and a Hopf bifurcation appears around $E_{★}=\left(S_{★},I_{★}\right)$ when $\tau =\tau_{0}.$*

We derived certain conditions in the preceding section under which system (20) experiences the Hopf bifurcation at $\tau =\tau_{0}.$ In this section, we assume that when $\tau =\tau_{0}$, system (20) experiences a Hopf bifurcation at the zero equilibrium, which is from the zero equilibrium, a family of periodic solutions bifurcates. In the following, we use the normal form theory and center manifold reduction from [53] to find the Hopf bifurcation direction, which determines whether the bifurcating branch of the periodic solution occurs locally for $\tau >\tau_{0}$ or $\tau <\tau_{0}$, and we identify the features of these bifurcating periodic solutions, such as the center manifold stability and period. It is important to assume in the following that $f\in C^{2}$. For convenience, let $u_{1}\left(t\right)=S\left(\tau t\right),u_{2}\left(t\right)=I\left(\tau t\right),\alpha =1$ and $\tau =\tau_{0}+q,$ where $\tau_{0}$ is defined in the above section and q∈R, then system (20) can be written as the functional differential equation in $C(\left[-1,0\right],R^{2})$ as

(45) $$\begin{array}{c} \dot{u}\left(t\right)=X_{q}\left(u_{t}\right)+G\left(q,u_{t}\right), \end{array}$$

where $u_{t}\left(r\right)=u\left(t+r\right)\in C\left(\left[-1,0\right],R^{2}\right)$, $X_{q}:C\left(\left[-1,0\right],R^{2}\right)\to R,$ and G:R×C([−1,0],$R^{2})\to R$ are, respectively, given by

$$\begin{array}{c} X_{q}\left(\theta \right)=\left(\tau_{0}+q\right)\left(\begin{array}{cc} -a_{1} & 0\\ a_{2} & -c_{1} \end{array}\right)\left(\begin{array}{c} \theta_{1}\left(0\right)\\ \theta_{2}\left(0\right) \end{array}\right)+\left(\tau_{0}+q\right)\left(\begin{array}{cc} 0 & -b_{1}\\ 0 & b_{2} \end{array}\right)\left(\begin{array}{c} \theta_{1}\left(-1\right)\\ \theta_{2}\left(-1\right) \end{array}\right), \end{array}$$

and

$$\begin{array}{c} G\left(q,\theta \right)=\left(\tau_{0}+q\right)\left(\begin{array}{c} m_{1}\theta_{1}^{2}\left(0\right)+m_{2}\theta_{2}^{2}\left(-1\right)+m_{3}\theta_{1}\left(0\right)\theta_{2}\left(-1\right)+m_{4}\theta_{1}^{3}\left(0\right)\\ +m_{5}\theta_{1}\left(0\right)\theta_{2}^{2}\left(-1\right)+m_{6}\theta_{1}^{2}\left(0\right)\theta_{2}\left(-1\right)+m_{7}\theta_{2}^{3}\left(-1\right)+h.o.t\\ n_{1}\theta_{1}^{2}\left(0\right)+n_{2}\theta_{2}^{2}\left(-1\right)+n_{3}\theta_{1}\left(0\right)\theta_{2}\left(-1\right)+n_{4}\theta_{1}^{3}\left(0\right)\\ +n_{5}\theta_{1}\left(0\right)\theta_{2}^{2}\left(-1\right)+n_{6}\theta_{1}^{2}\left(0\right)\theta_{2}\left(-1\right)+n_{7}\theta_{2}^{3}\left(-1\right)+h.o.t \end{array}\right), \end{array}$$

where $\theta \left(r\right)={\left(\theta_{1}\left(r\right),\theta_{2}\left(r\right)\right)}^{T}\in C\left(\left[-1,0\right],R^{2}\right),\;m_{1}=-n_{1}=\left(-\gamma \frac{\partial^{2}f_{1}\left(0,0\right)}{\partial u_{1}^{2}}\right)/2,\;m_{2}=-n_{2}=\left(-\gamma \frac{\partial^{2}f_{1}\left(0,0\right)}{\partial u_{2}^{2}}\right)/2,\;m_{3}=-n_{3}=\left(-\gamma \frac{\partial^{2}f_{1}\left(0,0\right)}{\partial u_{1}\partial u_{2}}\right),\;m_{4}=-n_{4}=\left(-\gamma \frac{\partial^{3}f_{1}\left(0,0\right)}{\partial u_{1}^{3}}\right)/6,\;m_{5}=-n_{5}=\left(-\gamma \frac{\partial^{3}f_{1}\left(0,0\right)}{\partial u_{1}\partial u_{2}^{2}}\right)/2,\;m_{6}=-n_{6}=\left(-\gamma \frac{\partial^{3}f_{1}\left(0,0\right)}{\partial u_{1}^{2}\partial u_{2}}\right)/2,\;m_{7}=-n_{7}=\left(-\gamma \frac{\partial^{3}f_{1}\left(0,0\right)}{\partial u_{2}^{3}}\right)/6$.

According to the discussions above, if q=0, system (45) experiences a Hopf bifurcation at the zero equilibrium, and the associated characteristic equation of system (45) has a pair of pure imaginary roots $\pm i\tau_{0}\omega_{0}$.

According to the Riesz representation theorem [54], there exists a bounded variation function φ(r,q) for r∈[−1,0], such that

(46) $$\begin{array}{c} X_{q}\left(\theta \right)=\int_{-1}^{0}d\varphi \left(r,0\right)\theta \left(r\right),\;\mathrm{for}\;\theta \in C\left(\left[-1,0\right],R^{2}\right). \end{array}$$

Moreover, one can choose

(47) $$\begin{array}{c} \varphi \left(r,q\right)=\left(\tau_{0}+q\right)\left(\begin{array}{cc} -a_{1} & 0\\ a_{2} & -c_{1} \end{array}\right)\phi \left(r\right)+\left(\tau_{0}+q\right)\left(\begin{array}{cc} 0 & -b_{1}\\ 0 & b_{2} \end{array}\right)\phi \left(r\right), \end{array}$$

where ϕ is defined by

(48) $$\begin{array}{c} \phi \left(r\right)=\left\{\begin{array}{cc} 0, & r\neq 0,\\ 1, & r=0. \end{array}\right. \end{array}$$

For $\theta \in C^{1}\left(\left[-1,0\right],R^{2}\right)$, define

(49) $$\begin{array}{c} L\left(q\right)\theta =\left\{\begin{array}{cc} \frac{d\theta (r)}{dr}, & r\in [-1,0),\\ \int_{-1}^{0}d\varphi \left(q,s\right)\theta \left(s\right), & r=0, \end{array}\right. \end{array}$$

and

(50) $$\begin{array}{c} M\left(q\right)\theta =\left\{\begin{array}{cc} 0, & r\in [-1,0),\\ G(q,\theta ), & r=0. \end{array}\right. \end{array}$$

The functional differential system (45) is then equivalent to

(51) $$\begin{array}{c} {\dot{u}}_{t}=L\left(q\right)u_{t}+M\left(q\right)u_{t} \end{array}$$

where $u_{t}\left(r\right)=u\left(t+r\right)$ for r∈[−1,0].

For $\vartheta \in C^{1}\left(\left[0,1\right],{\left(R^{2}\right)}^{*}\right)$, the adjoint operator $L^{*}\left(q\right)$ of L(q) is defined by

(52) $$\begin{array}{c} L^{*}\left(q\right)\vartheta \left(s\right)=\left\{\begin{array}{cc} -\frac{d\vartheta (s)}{ds}, & s\in (0,1],\\ \int_{-1}^{0}d\varphi^{T}\left(s,q\right)\vartheta \left(-s\right), & s=0. \end{array}\right. \end{array}$$

and a bilinear inner product for $\theta \in C^{1}\left(\left[-1,0\right],R^{2}\right)$, and $\vartheta \in C^{1}\left(\left[0,1\right],{\left(R^{2}\right)}^{*}\right)$ is defined by

(53) $$\begin{array}{c} \langle \vartheta \left(s\right),\theta \left(r\right)\rangle =\bar{\vartheta }\left(0\right)\theta \left(0\right)-\int_{r=-1}^{0}\int_{\eta =0}^{r}\bar{\vartheta }\left(\eta -r\right)d\varphi \left(r\right)\theta \left(\eta \right)d\eta , \end{array}$$

where φ(r)=φ(r,0). Then, L(0) and $L^{*}\left(0\right)$ are adjoint operators, as $\langle \vartheta \left(s\right),L\left(0\right)\theta \left(r\right)\rangle =\langle L^{*}\left(0\right)\vartheta \left(s\right),\theta \left(r\right)\rangle .$

From Lemma 4, we know that $\pm i\tau_{0}\omega_{0}$ are eigenvalues of L(0). As a result, they are also eigenvalues of $L^{*}\left(0\right)$. Suppose that $\kappa \left(r\right)={\left(1,e_{1}\right)}^{T}e^{ir\omega_{0}\tau_{0}}$ is the eigenvector of L(0) corresponding to the eigenvalue $i\tau_{0}\omega_{0}$. Then, $L\left(0\right)\kappa \left(r\right)=i\tau_{0}\omega_{0}\kappa \left(r\right).$ From (46) and (49), one has

(54) $$\begin{array}{c} \left\{\begin{array}{cc} L\left(0\right)\kappa \left(r\right)=\frac{d\kappa (r)}{dr}=i\tau_{0}\omega_{0}\kappa \left(r\right), & r\in [-1,0)\\ X_{q}\kappa \left(0\right)=i\tau_{0}\omega_{0}\kappa \left(0\right), & r=0. \end{array}\right. \end{array}$$

According to the definitions of L(0) and (47), where

(55) $$\begin{array}{c} \tau_{0}\left(\begin{array}{cc} a_{1}+i\omega_{0} & b_{1}e^{-i\omega_{0}\tau_{0}}\\ -a_{2} & c_{1}-b_{2}e^{-i\omega_{0}\tau_{0}}+i\omega_{0} \end{array}\right)\kappa \left(0\right)=\left(\begin{array}{c} 0\\ 0 \end{array}\right), \end{array}$$

which gives that

$$\begin{array}{c} \kappa \left(0\right)={\left(1,e_{1}\right)}^{T}={\left(1,\frac{a_{2}}{c_{1}+i\omega_{0}-b_{2}e^{-i\omega_{0}\tau_{0}}}\right)}^{T}. \end{array}$$

Similarly, it can be demonstrated that $\kappa^{*}\left(s\right)=D\left(1,e_{1}^{*}\right)e^{is\omega_{0}\tau_{0}}$ is the eigenvector of $L^{*}\left(0\right)$ corresponding to the eigenvalue $-i\omega_{0}\tau_{0},$ where $e_{1}^{*}=\frac{a_{2}e^{i\omega_{0}\tau_{0}}}{c_{1}-b_{2}-i\omega_{0}}$. Then, $L^{*}\left(0\right)\kappa^{*}\left(s\right)=-i\tau_{0}\omega_{0}\kappa^{*}\left(s\right).$ To ensure that $\langle \kappa^{*}\left(s\right),\kappa \left(r\right)\rangle =1$, the value of *D* must be determined. By (45), (46), and (54), one has

(56) $$\begin{array}{cc} \langle \kappa^{*}\left(s\right),\kappa \left(r\right)\rangle = & \bar{D}\left(1,{\bar{e}}_{1}^{*}\right){\left(1,e_{1}\right)}^{T}-\int_{r=-1}^{0}\int_{\eta =0}^{r}{\left({\bar{\kappa }}^{*}\right)}^{T}\left(\eta -r\right)d\varphi \left(r\right)\kappa \left(\eta \right)d\eta ,\\ = & \bar{D}\left(1,{\bar{e}}_{1}^{*}\right){\left(1,e_{1}\right)}^{T}-\int_{r=-1}^{0}\int_{\eta =0}^{r}\bar{D}\left(1,{\bar{e}}_{1}^{*}\right)e^{-i\left(\eta -r\right)\omega_{0}r_{0}}d\varphi \left(r\right){\left(1,e_{1}\right)}^{T}e^{i\eta \omega_{0}\tau_{0}}d\eta ,\\ = & \bar{D}\left(1+e_{1}{\bar{e}}_{1}^{*}-\left(1,{\bar{e}}_{1}^{*}\right)\int_{r=-1}^{0}d\varphi \left(r\right)re^{i\tau_{0}\omega_{0}r}{\left(1,e_{1}\right)}^{T}\right),\\ = & \bar{D}\left(1+e_{1}{\bar{e}}_{1}^{*}-b_{1}e_{1}\tau_{0}e^{-i\tau_{0}\omega_{0}}\right). \end{array}$$

Thus, in order to ensure $\langle \kappa^{*}\left(s\right),\kappa \left(r\right)\rangle =1,D$ can be chosen as $D=\frac{1}{1+e_{1}{\bar{e}}_{1}^{*}-b_{1}e_{1}\tau_{0}e^{-i\tau_{0}\omega_{0}}}.$

Next, we compute the coordinates describing the center manifold $C_{0}$ at q=0 using the same notations as before. Let $u_{t}$ be the solution of (45) when q=0. Then, define

(57) $$\begin{array}{c} K\left(t\right)=\langle \kappa^{*},u_{t}\rangle \;\mathrm{and}\;H\left(t,r\right)=u_{t}\left(r\right)-2Re\left(K\left(t\right)\kappa \left(r\right)\right). \end{array}$$

In the center manifold $C_{0}$, we obtain

(58) $$\begin{array}{c} H\left(t,r\right)=H(K\left(t\right),\bar{K}\left(t\right),r) \end{array}$$

where

$$\begin{array}{c} H\left(K\left(t\right),\bar{K}\left(t\right),r\right)=H_{30}\left(r\right)\frac{K{\left(t\right)}^{2}}{2}+H_{11}\left(r\right)K\left(t\right)\bar{K}\left(t\right)+H_{02}\left(r\right)\frac{\bar{K}{\left(t\right)}^{2}}{2}+H_{30}\left(r\right)\frac{K{\left(t\right)}^{3}}{6}+\ldots , \end{array}$$

where *K* and $\bar{K}$ are local coordinates for the center manifold $C_{0}$ in the direction of $\kappa^{*}$ and ${\bar{\kappa }}^{*}.$ Notice that *H* is real if the solution (45) $u_{t}$ is real and we consider only real solutions. For the solution $u_{t}$ in the center manifold $C_{0}$ and q=0, one has

(59) $$\begin{array}{cc} \dot{K}\left(t\right)= & \langle \kappa^{*},{\dot{u}}_{t}\rangle ,\\ = & \langle \kappa^{*},Lu_{t}+Mu_{t}\rangle ,\\ = & \langle \kappa^{*},Lu_{t}\rangle +\langle \kappa^{*},Mu_{t}\rangle ,\\ = & \langle L^{*}\kappa^{*},u_{t}\rangle +\langle \kappa^{*},Mu_{t}\rangle ,\\ = & \langle -i\tau_{0}\omega_{0}\kappa^{*},u_{t}\rangle +\langle \kappa^{*},Mu_{t}\rangle ,\\ = & i\tau_{0}\omega_{0}\left({\left({\bar{\kappa }}^{*}\left(0\right)\right)}^{T}u_{t}\left(0\right)-\int_{r=-1}^{0}\int_{\eta =0}^{r}{\left({\bar{\kappa }}^{*}\right)}^{T}\left(\eta -r\right)d\varphi \left(r\right)u_{t}\left(\eta \right)d\eta \right)+\langle \kappa^{*},Mu_{t}\rangle ,\\ = & i\tau_{0}\omega_{0}\langle \kappa^{*},u_{t}\rangle +\langle \kappa^{*},Mu_{t}\rangle ,\\ = & i\tau_{0}\omega_{0}K+\langle \kappa^{*}\left(r\right),G\left(0,H\left(K,\bar{K},r\right)+2Re\left(K\kappa \left(r\right)\right)\right)\rangle ,\\ = & i\tau_{0}\omega_{0}K+{\bar{\kappa }}^{*}\left(0\right)G\left(0,H\left(K,\bar{K},0\right)+2Re\left(K\kappa \left(0\right)\right)\right),\\ = & i\tau_{0}\omega_{0}K+{\bar{\kappa }}^{*}\left(0\right)G_{0}\left(K,\bar{K}\right). \end{array}$$

This equation is rewritten as

(60) $$\begin{array}{c} \dot{K}\left(t\right)=i\tau_{0}\omega_{0}K\left(t\right)+V\left(K,\bar{K}\right) \end{array}$$

where

(61) $$\begin{array}{c} V\left(K,\bar{K}\right)=V_{20}\frac{K{\left(t\right)}^{2}}{2}+V_{11}K\left(t\right)\bar{K}\left(t\right)+V_{02}\frac{\bar{K}{\left(t\right)}^{2}}{2}+V_{21}\frac{K{\left(t\right)}^{2}\bar{K}\left(t\right)}{2}+\ldots . \end{array}$$

From (57), we have $u_{t}\left(r\right)=\left(u_{1t}\left(r\right),u_{2t}\left(r\right)\right)=H\left(t,r\right)+K\kappa \left(r\right)+\bar{K}\bar{\kappa }\left(r\right)$ and $\kappa \left(r\right)={\left(1,e_{1}\right)}^{T}$$e^{ir\tau_{0}\omega_{0}}$ and then

$$\begin{array}{cc} u_{1t}\left(0\right)= & K+\bar{K}+H_{20}^{\left(1\right)}\left(0\right)\frac{K^{2}}{2}+H_{11}^{\left(1\right)}\left(0\right)K\bar{K}+H_{02}^{\left(1\right)}\left(0\right)\frac{{\bar{K}}^{2}}{2}+O(|K,\bar{K}{|}^{3}),\\ u_{21}\left(-1\right)= & Ke_{1}e^{-i\omega_{0}\tau_{0}}+\bar{K}{\bar{e}}_{1}e^{-i\omega_{0}\tau_{0}}+H_{20}^{\left(2\right)}\left(-1\right)\frac{K^{2}}{2}+H_{11}^{\left(2\right)}\left(-1\right)K\bar{K}+H_{02}^{\left(2\right)}\left(-1\right)\frac{{\bar{K}}^{2}}{2}+O(|K,\bar{K}{|}^{3}). \end{array}$$

It follows from the definition of G(t,r) that

(62) $$\begin{array}{cc} V(K,\bar{K})= & {\bar{\kappa }}^{*}\left(0\right)G_{0}\left(K,\bar{K}\right),\\ = & \bar{D}\tau_{0}\left(1,e_{1}\right)\left(\begin{array}{c} m_{1}u_{1t}^{2}\left(0\right)+m_{2}u_{2t}^{2}\left(-1\right)+m_{3}u_{1t}\left(0\right)u_{2t}\left(-1\right)+m_{4}u_{1t}^{3}\left(0\right)\\ +m_{5}u_{1t}\left(0\right)u_{2t}^{2}\left(-1\right)+m_{6}u_{1t}^{2}\left(0\right)u_{2t}\left(-1\right)+m_{7}u_{2t}^{3}\left(-1\right)+h.o.t\\ n_{1}u_{1t}^{2}\left(0\right)+n_{2}u_{2t}^{2}\left(-1\right)+n_{3}u_{1t}\left(0\right)u_{2t}\left(-1\right)+n_{4}u_{1t}^{3}\left(0\right)\\ +n_{5}u_{1t}\left(0\right)u_{2t}^{2}\left(-1\right)+n_{6}u_{1t}^{2}\left(0\right)u_{2t}\left(-1\right)+n_{7}u_{2t}^{3}\left(-1\right)+h.o.t \end{array}\right),\\ = & \bar{D}\tau_{0}\left\{\left({\bar{\gamma }}_{1}+{\bar{\gamma }}_{2}e_{1}^{2}e^{-2i\omega_{0}\tau_{0}}+{\bar{\gamma }}_{3}e_{1}e^{-i\omega_{0}\tau_{0}}\right)K^{2}+(2{\bar{\gamma }}_{1}+2{\bar{\gamma }}_{2}e_{1}{\bar{e}}_{1}e^{-2i\omega_{0}\tau_{0}}\right.\\ & +{\bar{\gamma }}_{3}e_{1}e^{-i\omega_{0}\tau_{0}}+{\bar{\gamma }}_{3}{\bar{e}}_{1}e^{-i\omega_{0}\tau_{0}})K\bar{K}+\left({\bar{\gamma }}_{1}+{\bar{\gamma }}_{2}{\bar{e}}_{1}^{2}e^{-2i\omega_{0}\tau_{0}}+{\bar{\gamma }}_{3}{\bar{e}}_{1}e^{-i\omega_{0}\tau_{0}}\right){\bar{K}}^{2}\\ & +(2{\bar{\gamma }}_{1}H_{11}^{\left(1\right)}\left(0\right)+{\bar{\gamma }}_{1}H_{20}^{\left(1\right)}\left(0\right)+2{\bar{\gamma }}_{2}e_{1}H_{11}^{\left(2\right)}\left(-1\right)e^{-i\omega_{0}\tau_{0}}+{\bar{\gamma }}_{2}{\bar{e}}_{1}H_{20}^{\left(2\right)}\left(-1\right)e^{-i\omega_{0}\tau_{0}}\\ & +{\bar{\gamma }}_{3}e_{1}H_{11}^{\left(1\right)}\left(0\right)e^{-i\omega_{0}\tau_{0}}+\frac{1}{2}{\bar{\gamma }}_{3}{\bar{e}}_{1}H_{20}^{\left(1\right)}\left(0\right)e^{-i\omega_{0}\tau_{0}}+{\bar{\gamma }}_{3}H_{11}^{\left(2\right)}\left(-1\right)+\frac{1}{2}{\bar{\gamma }}_{3}H_{20}^{\left(2\right)}\left(-1\right)\\ & +3{\bar{\gamma }}_{4}+{\bar{\gamma }}_{5}e_{1}^{2}e^{-2i\omega_{0}\tau_{0}}+2{\bar{\gamma }}_{5}e_{1}{\bar{e}}_{1}e^{-2i\omega_{0}\tau_{0}}+2{\bar{\gamma }}_{6}e_{1}e^{-i\omega_{0}\tau_{0}}+{\bar{\gamma }}_{6}{\bar{e}}_{1}e^{-i\omega_{0}\tau_{0}}\\ & \left.+3{\bar{\gamma }}_{7}e_{1}^{2}{\bar{e}}_{1}e^{-3i\omega_{0}\tau_{0}})K^{2}\bar{K}\right\} \end{array}$$

when we compare the coefficients to (61), we have

(63) $$\begin{array}{cc} V_{20}= & 2\bar{D}\tau_{0}\left({\bar{\gamma }}_{1}+{\bar{\gamma }}_{2}e_{1}^{2}e^{-2i\omega_{0}\tau_{0}}+{\bar{\gamma }}_{3}e_{1}e^{-i\omega_{0}\tau_{0}}\right),\\ V_{11}= & \bar{D}\tau_{0}\left(2{\bar{\gamma }}_{1}+2{\bar{\gamma }}_{2}e_{1}{\bar{e}}_{1}e^{-2i\omega_{0}\tau_{0}}+{\bar{\gamma }}_{3}e_{1}e^{-i\omega_{0}\tau_{0}}+{\bar{\gamma }}_{3}{\bar{e}}_{1}e^{-i\omega_{0}\tau_{0}}\right),\\ V_{02}= & 2\bar{D}\tau_{0}\left({\bar{\gamma }}_{1}+{\bar{\gamma }}_{2}{\bar{e}}_{1}^{2}e^{-2i\omega_{0}\tau_{0}}+{\bar{\gamma }}_{3}{\bar{e}}_{1}e^{-i\omega_{0}\tau_{0}}\right),\\ V_{21}= & (2{\bar{\gamma }}_{1}H_{11}^{\left(1\right)}\left(0\right)+{\bar{\gamma }}_{1}H_{20}^{\left(1\right)}\left(0\right)+2{\bar{\gamma }}_{2}e_{1}H_{11}^{\left(2\right)}\left(-1\right)e^{-i\omega_{0}\tau_{0}}+{\bar{\gamma }}_{2}{\bar{e}}_{1}H_{20}^{\left(2\right)}\left(-1\right)e^{-i\omega_{0}\tau_{0}}\\ & +{\bar{\gamma }}_{3}e_{1}H_{11}^{\left(1\right)}\left(0\right)e^{-i\omega_{0}\tau_{0}}+\frac{1}{2}{\bar{\gamma }}_{3}{\bar{e}}_{1}H_{20}^{\left(1\right)}\left(0\right)e^{-i\omega_{0}\tau_{0}}+{\bar{\gamma }}_{3}H_{11}^{\left(2\right)}\left(-1\right)+\frac{1}{2}{\bar{\gamma }}_{3}H_{20}^{\left(2\right)}\left(-1\right)\\ & +3{\bar{\gamma }}_{4}+{\bar{\gamma }}_{5}e_{1}^{2}e^{-2i\omega_{0}\tau_{0}}+2{\bar{\gamma }}_{5}e_{1}{\bar{e}}_{1}e^{-2i\omega_{0}\tau_{0}}+2{\bar{\gamma }}_{6}e_{1}e^{-i\omega_{0}\tau_{0}}+{\bar{\gamma }}_{6}{\bar{e}}_{1}e^{-i\omega_{0}\tau_{0}}\\ & +3{\bar{\gamma }}_{7}e_{1}^{2}{\bar{e}}_{1}e^{-3i\omega_{0}\tau_{0}}). \end{array}$$

In order to calculate $V_{21}$, we must first compute $H_{20}\left(r\right)$ and $H_{11}\left(r\right)$. By (51) and (57), one has

(64) $$\begin{array}{cc} \dot{H}={\dot{u}}_{t}-\dot{K}\kappa -\bar{\dot{K}\;\kappa }= & \left\{\begin{array}{cc} LH-2Re({\bar{\kappa }}^{*}\left(0\right)G_{0}\kappa \left(r\right)), & r\in [-1,0),\\ LH-2Re\left({\bar{\kappa }}^{*}\left(0\right)G_{0}\kappa \left(0\right)\right)+G_{0}, & r=0, \end{array}\right.\\ = & LH+Z(K,\bar{K},r), \end{array}$$

where

(65) $$\begin{array}{c} Z\left(K,\bar{K},r\right)=Z_{20}\left(r\right)\frac{K^{2}}{2}+Z_{11}\left(r\right)K\bar{K}+Z_{02}\left(r\right)\frac{{\bar{K}}^{2}}{2}+\ldots . \end{array}$$

and $G_{0}$ denotes $G(K,\bar{K})$ at q=0. In light of (65), one obtains

(66) $$\begin{array}{c} LH-\dot{H}=-Z\left(K,\bar{K},r\right). \end{array}$$

On the other hand, notice that on the center manifold $C_{0}$

(67) $$\begin{array}{c} \dot{H}=H_{20}\left(r\right)K\dot{K}+H_{11}\left(r\right)\left(\dot{K}\bar{K}+K\bar{\dot{K}}\right)+H_{02}\bar{K\dot{K}}+\ldots . \end{array}$$

This, along with (58) and (66), equals

(68) $$\begin{array}{c} \left(L-2i\omega_{0}\tau_{0}\right)H_{20}\left(r\right)=-Z_{20}\left(r\right),LH_{11}\left(r\right)=-Z_{11}\left(r\right),\left(L+2i\omega_{0}\tau_{0}\right)H_{02}\left(r\right)=-Z_{02}\left(r\right),\ldots . \end{array}$$

From (64), we know that for r∈[−1,0)

(69) $$\begin{array}{cc} Z(K,\bar{K},r)= & -{\bar{\kappa }}^{*}\left(0\right)G_{0}\left(K,\bar{K}\right)\kappa \left(r\right)-\kappa^{*}\left(0\right){\bar{G}}_{0}\left(K,\bar{K}\right)\bar{\kappa }\left(r\right),\\ = & -V\left(K,\bar{K}\right)\kappa \left(r\right)-\bar{V}\left(K,\bar{K}\right)\bar{\kappa }\left(r\right). \end{array}$$

When the coefficients are compared to (65), it is revealed that

(70) $$\begin{array}{c} \left\{\begin{array}{cc} Z_{20}\left(r\right)=-V_{20}\kappa \left(r\right)-{\bar{V}}_{02}\bar{\kappa }\left(r\right), & r\in [-1,0),\\ Z_{11}\left(r\right)=-V_{11}\kappa \left(r\right)-{\bar{V}}_{11}\bar{\kappa }\left(r\right), & r\in [-1,0). \end{array}\right. \end{array}$$

From (49), (68), and (70), we can obtain

(71) $$\begin{array}{c} {\dot{H}}_{20}\left(r\right)=2i\omega_{0}\tau_{0}H_{20}\left(r\right)+V_{20}\kappa \left(r\right)+{\bar{V}}_{02}\bar{\kappa }\left(r\right). \end{array}$$

We know that $\kappa \left(r\right)={\left(1,e_{1}\right)}^{T}e^{ir\omega_{0}\tau_{0}}$, one has

(72) $$\begin{array}{c} H_{20}\left(r\right)=\frac{iV_{20}}{\omega_{0}\tau_{0}}\kappa \left(0\right)e^{ir\omega_{0}\tau_{0}}+\frac{i{\bar{V}}_{02}}{3\omega_{0}\tau_{0}}\bar{\kappa }\left(0\right)e^{-ir\omega_{0}\tau_{0}}+P_{1}e^{2ir\omega_{0}\tau_{0}} \end{array}$$

where $P_{1}=\left(P_{1}^{\left(1\right)},P_{1}^{\left(2\right)}\right)\in R^{2}$ is a constant vector. Similarly, we can obtain from (68) and (70)

(73) $$\begin{array}{c} H_{11}\left(r\right)=-\frac{iV_{11}}{\omega_{0}\tau_{0}}\kappa \left(0\right)e^{ir\omega_{0}\tau_{0}}+\frac{i{\bar{V}}_{11}}{\omega_{0}\tau_{0}}\bar{\kappa }\left(0\right)e^{-ir\omega_{0}\tau_{0}}+P_{2} \end{array}$$

where $P_{2}=\left(P_{2}^{\left(1\right)},P_{2}^{\left(2\right)}\right)\in R^{2}$ is also a constant vector.

Following that, $P_{1}$ and $P_{2}$ will be determined. Based on the definition of *L* and (68),

(74) $$\begin{array}{c} \int_{r=1}^{0}d\varphi \left(r\right)H_{20}\left(r\right)=2i\omega_{0}\tau_{0}H_{20}\left(0\right)-Z_{20}\left(0\right) \end{array}$$

and

(75) $$\begin{array}{c} \int_{-1}^{0}d\varphi \left(r\right)H_{11}\left(r\right)=-Z_{11}\left(0\right), \end{array}$$

where φ(r)=φ(0,r). By (64) and (65), we have

(76) $$\begin{array}{cc} Z(K,\bar{K},0)= & -2Re\left({\bar{\kappa }}^{*}\left(0\right)G_{0}\left(K,\bar{K}\right)\kappa \left(0\right)\right)+G_{0}\left(K,\bar{K}\right),\\ = & -V\left(K,\bar{K}\right)\kappa \left(0\right)-\bar{V}\left(K,\bar{K}\right)\bar{\kappa }\left(0\right)+G_{0}\left(K,\bar{K}\right). \end{array}$$

When the coefficients of (76) and (65) are compared, the result is

(77) $$\begin{array}{c} Z_{20}\left(0\right)=-V_{20}\kappa \left(0\right)-{\bar{V}}_{02}\bar{\kappa }\left(0\right)+2\tau_{0}\left(\begin{array}{c} m_{1}+m_{2}e^{-2i\omega_{0}\tau_{0}}e_{1}^{2}+m_{3}e^{-i\omega_{0}\tau_{0}}e_{1}\\ n_{1}+n_{2}e^{-2i\omega_{0}\tau_{0}}e_{1}^{2}+n_{3}e^{-i\omega_{0}\tau_{0}}e_{1} \end{array}\right) \end{array}$$

and

(78) $$\begin{array}{c} Z_{11}\left(0\right)=-V_{11}\kappa \left(0\right)-{\bar{V}}_{11}\bar{\kappa }\left(0\right)+2\tau_{0}\left(\begin{array}{c} 2m_{1}+2e^{-2i\omega_{0}\tau_{0}}\left|e_{1}\right|m_{2}+e^{-i\omega_{0}\tau_{0}}m_{3}\left({\bar{e}}_{1}+e_{1}\right)\\ 2n_{1}+2e^{-2i\omega_{0}\tau_{0}}\left|e_{1}\right|n_{2}+e^{-i\omega_{0}\tau_{0}}n_{3}\left({\bar{e}}_{1}+e_{1}\right) \end{array}\right). \end{array}$$

By noticing that

(79) $$\begin{array}{c} \left(i\omega_{0}\tau_{0}I-\int_{r=1}^{0}e^{ir\omega_{0}\tau_{0}}d\varphi \left(r\right)\right)\kappa \left(0\right)=0\;\mathrm{and}\;\left(-i\omega_{0}\tau_{0}I-\int_{r=1}^{0}e^{-ir\omega_{0}\tau_{0}}d\varphi \left(r\right)\right)\bar{\kappa }\left(0\right)=0 \end{array}$$

then form (72) and (77), we obtain

(80) $$\begin{array}{c} \left(2i\omega_{0}\tau_{0}I-\int_{r=1}^{0}e^{2ir\omega_{0}\tau_{0}}d\varphi \left(r\right)\right)P_{1}=2\tau_{0}\left(\begin{array}{c} m_{1}+m_{2}e^{-2i\omega_{0}\tau_{0}}e_{1}^{2}+m_{3}e^{-i\omega_{0}\tau_{0}}e_{1}\\ n_{1}+n_{2}e^{-2i\omega_{0}\tau_{0}}e_{1}^{2}+n_{3}e^{-i\omega_{0}\tau_{0}}e_{1} \end{array}\right) \end{array}$$

where

$$\begin{array}{c} 2i\omega_{0}\tau_{0}I-\int_{r=1}^{0}e^{2ir\omega_{0}\tau_{0}}d\varphi \left(r\right)=\tau_{0}\left(2i\omega_{0}I-\left(\begin{array}{cc} -a_{1} & 0\\ a_{2} & -c_{1} \end{array}\right)-\left(\begin{array}{cc} 0 & -b_{1}\\ 0 & b_{2} \end{array}\right)e^{-2i\tau_{0}\omega_{0}}\right) \end{array}$$

which leads to

$$\begin{array}{c} \left(\begin{array}{cc} 2i\omega_{0}+a_{1} & b_{1}e^{-2i\omega_{0}\tau_{0}}\\ a_{2} & 2i\omega_{0}+c_{1}-b_{2}e^{-2i\omega_{0}\tau_{0}} \end{array}\right)P_{1}=2\left(\begin{array}{c} m_{1}+m_{2}e^{-2i\omega_{0}\tau_{0}}e_{1}^{2}+m_{3}e^{-i\omega_{0}\tau_{0}}e_{1}\\ n_{1}+n_{2}e^{-2i\omega_{0}\tau_{0}}e_{1}^{2}+n_{3}e^{-i\omega_{0}\tau_{0}}e_{1} \end{array}\right). \end{array}$$

We obtain

(81) $$\begin{array}{c} P_{1}^{\left(1\right)}=\frac{\Delta_{1}}{\Delta_{2}},P_{1}^{\left(2\right)}=\frac{\Delta_{3}}{\Delta_{4}} \end{array}$$

where

$$\begin{array}{cc} \Delta_{1}= & -2e_{1}^{2}\left(b_{2}m_{2}+b_{1}n_{2}\right)-2e^{i\omega_{0}\tau_{0}}e_{1}\left(b_{2}m_{3}+b_{1}n_{3}\right)+2e^{4i\omega_{0}\tau_{0}}m_{1}\left(c_{1}+2i\omega_{0}\right)\\ & +2e^{3i\omega_{0}\tau_{0}}e_{1}m_{3}\left(c_{1}+2i\omega_{0}\right)-2e^{2i\omega_{0}\tau_{0}}\left(b_{2}m_{1}+b_{1}n_{1}-e_{1}^{2}m_{2}\left(c_{1}+2i\omega_{0}\right)\right),\\ \Delta_{2}= & e^{4i\omega_{0}\tau_{0}}\left(a_{1}+2i\omega_{0}\right)\left(c_{1}+2i\omega_{0}\right)-e^{2i\omega_{0}\tau_{0}}\left(a_{2}b_{1}+b_{2}\left(a_{1}+2i\omega_{0}\right)\right),\\ \Delta_{3}= & -2a_{2}\left(e^{2i\omega_{0}\tau_{0}}m_{1}+e_{1}\left(e_{1}m_{2}+e^{i\omega_{0}\tau_{0}}m_{3}\right)\right)+2\left(e^{2i\omega_{0}\tau_{0}}n_{1}+e_{1}\left(e_{1}n_{2}+e^{i\omega_{0}\tau_{0}}n_{3}\right)\right)\left(a_{1}+2i\omega_{0}\right),\\ \Delta_{4}= & -a_{2}b_{1}+\left(a_{1}+2i\omega_{0}\right)\left(-b_{2}+e^{2i\omega_{0}\tau_{0}}\left(c_{1}+2i\omega_{0}\right)\right). \end{array}$$

Similarly, by substituting (73) and (78) into (75), we can obtain

(82) $$\begin{array}{c} \left(\begin{array}{cc} a_{1} & b_{1}\\ -a_{2} & c_{1}-b_{2} \end{array}\right)P_{2}=2\left(\begin{array}{c} 2m_{1}+2e^{-2i\omega_{0}\tau_{0}}\left|e_{1}\right|m_{2}+e^{-i\omega_{0}\tau_{0}}m_{3}\left({\bar{e}}_{1}+e_{1}\right)\\ 2n_{1}+2e^{-2i\omega_{0}\tau_{0}}\left|e_{1}\right|n_{2}+e^{-i\omega_{0}\tau_{0}}n_{3}\left({\bar{e}}_{1}+e_{1}\right) \end{array}\right) \end{array}$$

and we have

(83) $$\begin{array}{c} P_{2}^{\left(1\right)}=\frac{\Delta_{5}}{a_{2}b_{1}+a_{1}\left(-b_{2}+c_{1}\right)},P_{2}^{\left(2\right)}=\frac{\Delta_{6}}{a_{2}b_{1}+a_{1}\left(-b_{2}+c_{1}\right)} \end{array}$$

where

$$\begin{array}{cc} \Delta_{5}= & e^{-2i\omega_{0}\tau_{0}}(-4e^{2i\omega_{0}\tau_{0}}\left(\left(b_{2}-c_{1}\right)m_{1}+b_{1}n_{1}\right)-4\left|e_{1}\right|\left(\left(b_{2}-c_{1}\right)m_{2}+b_{1}n_{2}\right)\\ & -2e^{i\omega_{0}\tau_{0}}\left(e_{1}+{\bar{e}}_{1}\right)\left(\left(b_{2}-c_{1}\right)m_{3}+b_{1}n_{3}\right)),\\ \Delta_{6}= & e^{-2i\omega_{0}\tau_{0}}(4e^{2i\omega_{0}\tau_{0}}\left(a_{2}m_{1}+a_{1}n_{1}\right)+4|e_{1}\left|\left(a_{2}m_{2}+a_{1}n_{2}\right)+2e^{i\omega_{0}\tau_{0}}\left(e_{1}+{\bar{e}}_{1}\right)\left(a_{2}m_{3}+a_{1}n_{3}\right)\right). \end{array}$$

Further, we can also calculate the values listed below:

$$\begin{array}{cc} \beta_{2}= & 2Re(\frac{i}{2\omega_{0}\tau_{0}}\left(V_{11}V_{20}-2{\left|V_{11}\right|}^{2}-\frac{|V_{02}{|}^{2}}{3}\right)+\frac{V_{21}}{2}),\\ k_{2}= & -\frac{Re(\frac{i}{2\omega_{0}\tau_{0}}\left(V_{11}V_{20}-2{\left|V_{11}\right|}^{2}-\frac{|V_{02}{|}^{2}}{3}\right)+\frac{V_{21}}{2})}{Re(\lambda^{{}^{\prime }}\left(\tau_{0}\right))},\\ T_{2}= & -\frac{Im\left(\frac{i}{2\omega_{0}\tau_{0}}\left(V_{11}V_{20}-2{\left|V_{11}\right|}^{2}-\frac{|V_{02}{|}^{2}}{3}\right)+\frac{V_{21}}{2}\right)+k_{2}Im\left(\lambda^{{}^{\prime }}\left(\tau_{0}\right)\right)}{\tau_{0}\omega_{0}} \end{array}$$

that establishes the number of bifurcating periodic solutions on the center manifold at $\tau_{0}$. Then the following results are obtained

**Theorem** **3.**

(*i*) *The stability of the bifurcating periodic solution is determined by $\beta_{2}$: when $\beta_{2}<0\;or\;\left(\beta_{2}>0\right)$, the bifurcating periodic solutions are stable or (unstable).*
(*ii*) *The direction of the Hopf bifurcation is determined by $k_{2}$: when $k_{2}>0$ or $\left(k_{2}<0\right)$, the Hopf bifurcation is supercritical (subcritical), and for $\tau >\tau_{0}\left(\tau <\tau_{0}\right)$, bifurcating periodic solutions exist.*
(*iii*) *The period of the bifurcating periodic solution is determined by $T_{2}$: when $T_{2}>0\left(T_{2}<0\right)$, the period increases (decreases).*

## 4. Simulation Results and Discussion

This section numerically investigates the local stability and Hopf bifurcation of the COVID-19 vaccination model, exhibiting our findings from Section 4. Moreover, we simulate how vaccinations impact COVID-19 prevention and control. Finally, we investigate the impact of time delay on the epidemic, and we make reasonable suggestions for effectively reducing the COVID-19 epidemic. All numerical computations were carried out in MATLAB R2020b and Maple 2013 numerical computing environments using the Adams–Bashforth method. Calculating the parameters of the model is difficult because the COVID-19 scenario changes frequently and from nation to nation. The parameters are likely to change over time as new policies are implemented on a daily basis. As a result, in order to simulate the COVID-19 vaccination model (2), we use certain model parameters from the literature and estimate or assume the rest based on actual conditions. For other assumed values, the model is stable and can provide the model results under reasonable conditions. The most extensively used vaccinations currently available have efficacies of 95% (Pfizer) for the COVID-19 mRNA vaccine BNT162b2, 94.1% (Moderna) for the mRNA-1273 vaccine, 78% for Sinovac, and 70.4% (AstraZeneca) for the ChAdOx1 nCoV-19 vaccine/AZD1222, according to reports from appropriate departments. We take ν∈[0.8,0.9] by considering the efficacy of various vaccines.

**Figure 2:**
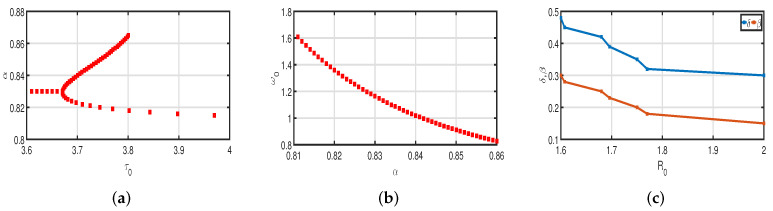
(**a**) Bifurcating parameter $\tau_{0}$. (**b**) Critical frequency $\omega_{0}$. (**c**) Basic reproduction number $R_{0}$.

In Table 1, all parameter values are displayed. These factors are used to calculate $R_{0}$ without vaccinations, as shown in Table 2 and Figure 2c. This shows that during the infection period, a COVID-19-infected individual can cause disease, on average, 2 to 3 susceptible individuals. In this instance, COVID-19 is spreading quickly. In this model, stability can be achieved and valid conclusions can be drawn with reasonable parameter assumptions.

**Figure 3:**
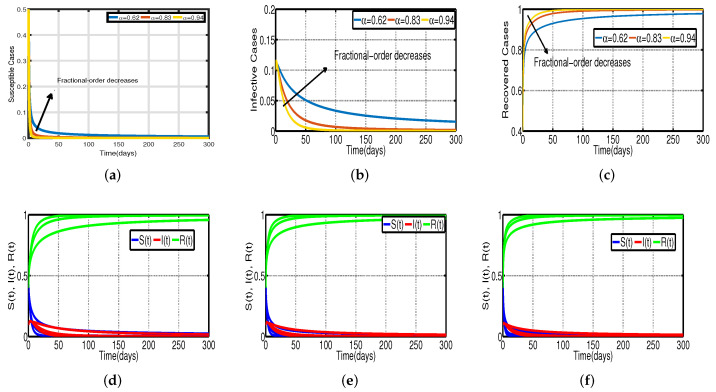
Vaccination model (2) without time delay. (**a**) Susceptible S(t). (**b**) Infected I(t). (**c**) Recovered R(t). (**d**) Vaccination rate of 0.5%. (**e**) Vaccination rate of 0.8%. (**f**) Vaccination rate of 1.2%.

**Figure 4:**
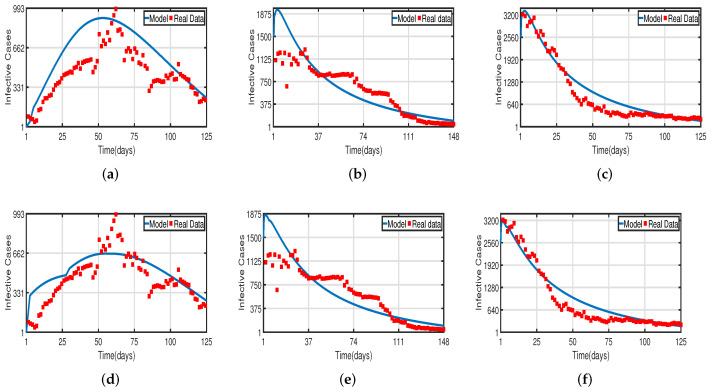
Fitting infected cases of model (2) for COVID-19 versus real observations of infected cases in the UAE. (**a**) Model fitting without the vaccinated population. (**b**) Model fitting with the vaccinated (after 50%) population. (**c**) Model fitting with the vaccinated (after 100%) population. (**d**) Time-delayed model fitting without the vaccinated population. (**e**) Time-delayed model fitting with the vaccinated (after 50%) population. (**f**) Time-delayed model fitting with the vaccinated (after 100%) population.

In Figure 3, the vaccination model (2) is presented without time delay. The effect on the population’s susceptible S(t), infected I(t), and recovered R(t) cases can be seen; whereas Figure 3d–f show that the proportion of susceptible individuals, S(t), declines as vaccination rates, $V_{ac}$, rise, and the proportion of recovered individuals, R(t), increases. With the chosen fixed vaccination efficacy rate ν, two significant points were obtained, one is model (2) with the vaccination percentage $V_{ac}=0.5\%$; it is evident that Figure 4d shows that the percentage of the spread of the disease decreases. In model (2) with $V_{ac}=1.2\%$, it is evident that Figure 4f shows that the percentage of the spread of the disease further decreases. This implies that the virus will stop spreading more quickly with a higher vaccination rate.

**Figure 5:**
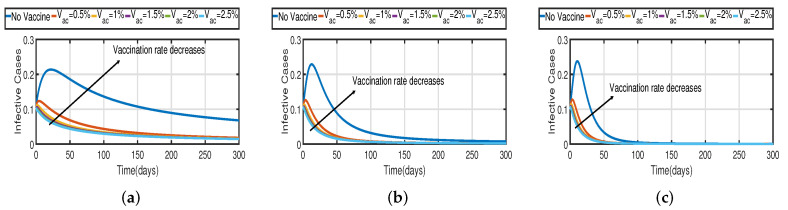
The trend of infected individuals with respect to different vaccination rates $V_{ac}.$ (**a**) Fractional-order α=0.62. (**b**) Fractional-order α=0.83. (**c**) Fractional-order α=0.94.

Taking the initial state value as I(t)=100, we analyze the behaviors of the numerous different infected populations I(t) without time delay with a fixed efficacy rate ν within 300 days in the two vaccination cases (with and without), as shown in Figure 5. It can be seen that the level of infectiousness decreases as the vaccination rate $V_{ac}$ increases from 0.5% to 2.5%. This demonstrates that COVID-19 can be effectively contained through vaccination. Figure 5a shows the changing trend of the infected population with fractional-order α=0.62. Without vaccination, the infected population rises at around the 30th day and there are about 220 infected individuals. The peak of the infected population I(t) in the case of the vaccination rate $\left(V_{ac}=0.5\%\right)$ occurs around day 15, and there are roughly 135 asymptomatic individuals. In Figure 5b,c the peak of the infected population I(t) in the case of the vaccination rate $\left(V_{ac}=0.5\%\right)$ occurs around day 12 and day 8, and there are roughly 137 and 139 asymptomatic individuals, respectively. That is, a 7-day delay in the peak of I(t) will significantly lessen the burden that COVID-19 is placing on medical resources, allowing more people to access timely medical care and lowering mortality.

**Figure 6:**
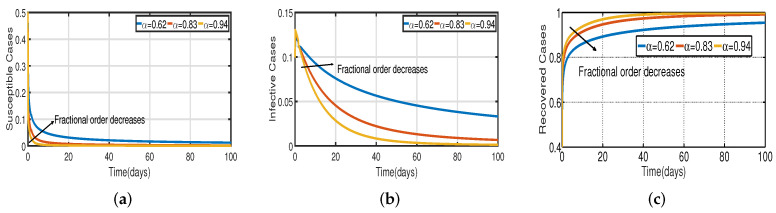
Vaccination model (2) with time delay $\tau =1.5\in [0,\tau_{0}).$ (**a**) Susceptible S(t). (**b**) Infected I(t). (**c**) Recovered R(t).

**Figure 7:**
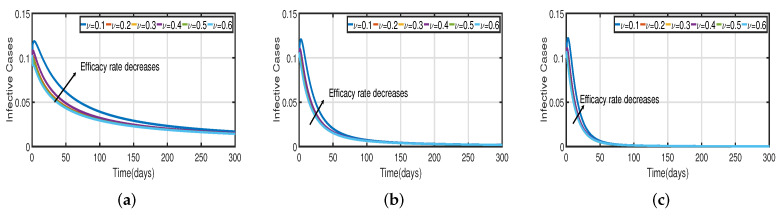
The trend of infected individuals regarding different vaccination efficacy rates ν. (**a**) Fractional-order α=0.62. (**b**) Fractional-order α=0.83. (**c**) Fractional-order α=0.94.

In Figure 6, the vaccination model (2) is presented with a time delay. The effect on the population’s susceptible S(t), infected I(t), and recovered R(t) cases can be seen; whereas the proportion of susceptible individuals S(t) declines as the fractional order α increases, while the proportion of recovered individuals R(t) declines with the fixed vaccination rate $V_{ac}=0.5\%.$ From the analysis of Figure 5, Figure 6b and Figure 7, it is evident that increased vaccination doses can minimize and delay the peak of infection to a greater extent. Thus, improving vaccination efficacy can help to prevent the spread of COVID-19 more effectively. However, in practice, the effectiveness of vaccines cannot be improved quickly, and COVID-19 cannot be quickly controlled by simply increasing vaccination rates. Therefore, in addition to vaccinations, several non-pharmaceutical measures must be used. Figure 5a and Figure 6b compare the trends in the number of affected individuals for the vaccination rate $V_{ac}=0.5\%$ and fractional order α=0.62, with a rate ν within 100 days. The peak of the infected population I(t) in the case of a vaccination rate of $\left(V_{ac}=0.5\%\right)$ occurs around day 15 (Figure 5a) and day 2 (Figure 6b), with roughly 135 and 130 asymptomatic individuals, respectively.

**Figure 8:**
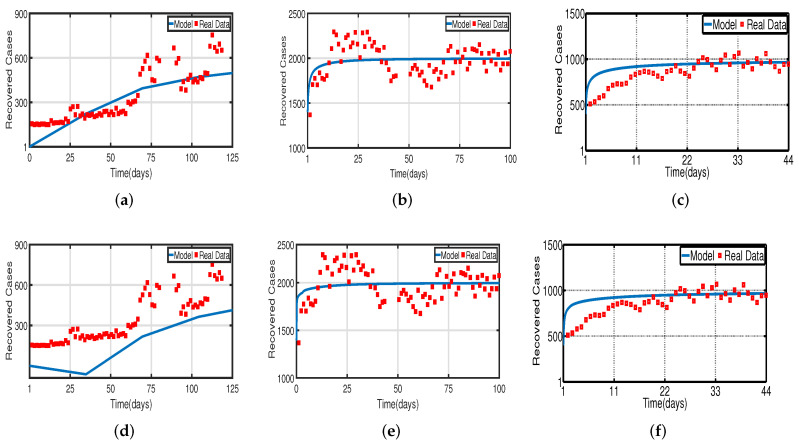
Fitting the recovered cases of model (2) for COVID-19 versus real observations of recovered cases in the UAE. (**a**) Model fitting without the vaccinated population. (**b**) Model fitting with the vaccinated (after 50%) population. (**c**) Model fitting with the vaccinated (after 100%) population. (**d**) Time-delayed model fitting without the vaccinated population. (**e**) Time-delayed model fitting with the vaccinated (after 50%) population. (**f**) Time-delayed model fitting with the vaccinated (after 100%) population.

Daily COVID-19 cases in the UAE were considered for the time period from 25 March to 30 July 2020 for the population without vaccination, from 15 July 2021 to 15 December 2021 for a 50% vaccinated population, and from 15 January 2022 to 20 May 2022 for a 100% vaccinated population. The daily COVID-19 updates were obtained from [55]. The graphical data in Figure 4 and Figure 8 illustrate the evolution of diagnosed infected and recovered cases with and without delays, demonstrating the biological impact of delay parameters. We fit model (2) to daily new infected cases of COVID-19 in the UAE from 25 March to 30 July 2020 in Figure 4a,d and fit model (2) to daily new recovered cases of COVID-19 for the UAE in Figure 8a,d. Figure 4b,e, show the fitting of model (2) to the cumulative daily COVID-19 vaccination data in the UAE from 15 July 2021 to 15 December 2021 for a 50% vaccinated population, and in Figure 8b,e, we fit model (2) to the recovered cases after 50% of the UAE population was vaccinated. In Figure 4c,f, the model is fitted to the cumulative daily COVID-19 vaccination data in the UAE from 15 January 2022 to 20 May 2022 for 100%, and in Figure 8c,f, we fit the model to the recovered cases after 100% of the UAE population was vaccinated. The number of diagnosed infected and recovered cases is highly impacted by delays, as seen in Figure 4 and Figure 8. As a result, the plot of model (2) without delays is different from that of the clinical data. Thus, we can conclude that delays are crucial to understanding the dynamic behavior of COVID-19 around the world, particularly in the UAE. The two figures, Figure 4 and Figure 8, individually demonstrate how well our model fits the three data sets from [55]. Therefore, our vaccine efficacy models are efficient in describing the spread of COVID-19 in the UAE.

**Figure 9:**
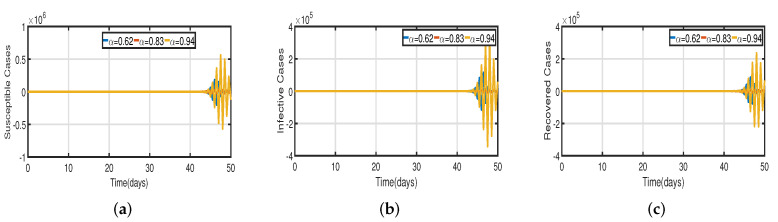
Vaccination model (2) with time delay $\tau >\tau_{0}.$ (**a**) Susceptible S(t). (**b**) Infected I(t). (**c**) Recovered R(t).

**Figure 10:**
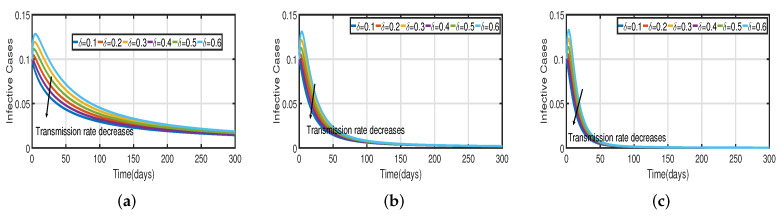
The trend of infected individuals regarding different transmission rates δ with a vaccination rate of 0.5%. (**a**) Fractional-order α=0.62. (**b**) Fractional-order α=0.83. (**c**) Fractional-order α=0.94.

**Figure 11:**
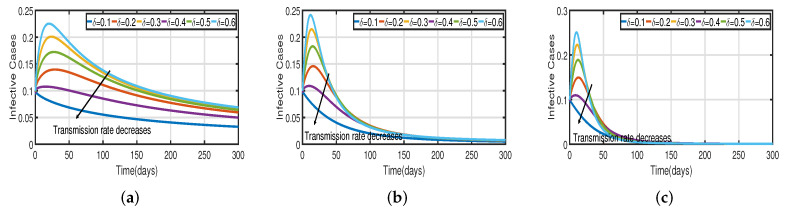
The trend of infected individuals regarding different transmission rates δ without a vaccination rate. (**a**) Fractional-order α=0.62. (**b**) Fractional-order α=0.83 (**c**) Fractional-order α=0.94.

Figure 3 and Figure 5 demonstrate that vaccination ($V_{ac}$) is effective in lowering infection rates and that early treatment and management of COVID-19 have a positive impact. It is also clear that despite the high efficiency of COVID-19 vaccines, the outbreak was difficult to control. One of the reasons for this is the occurrence of bifurcation, where I(t) converges to a non-zero constant. In this instance, COVID-19 coexists with humans for a considerable amount of time before becoming an endemic disease. If I(t) converges to zero, the rate of convergence is quite slow since the basic reproduction number is too large and close to one. We need to perform a calculation to determine the bifurcating parameter $\tau_{0}$ that causes this. It is not difficult to obtain the bifurcating parameter $\tau_{0}$ and the critical frequency $\omega_{0}$ (see Table 3 and Figure 2b). Moreover, the bifurcating parameter varies according to model parameters such as the transmission rate δ, recovery rate β (see Table 2), vaccination rate $V_{ac}$ (see Table 4), and efficacy rate ν (see Table 5). Since $\tau >\tau_{0}$, bifurcation appears in the vaccination model (2), as shown in Figure 2a (the curve corresponding to the fractional-order α) and Figure 9. We compare the dynamics of the number of infected cases without time delay with different transmission rates δ within 300 days (see Figure 10) for with and without vaccination (see Figure 11). It can be seen that the level of infectiousness decreases as the vaccination rate $V_{ac}=0.5\%$ if fixed. This demonstrates that COVID-19 can be effectively contained by increasing the vaccination rate $V_{ac}$.

Recent research has focused on investigating the long-term effects of vaccination on controlling COVID-19 incidence, utilizing mathematical modeling as seen in [56,57]. In biological systems with memory, both time delays and fractional orders play a significant role, which provides the model with greater flexibility. To investigate the impact of vaccination coverage on disease incidence, we simulate the COVID-19 fractional-order time-delayed vaccine model (2). Figure 5 and Figure 7 present comparison plots for the Caputo fractional-order model, considering various values of the fractional-order parameter α=0.62,0.83,0.94; the graphical results are presented for comparison. The number of infected individuals decreases as vaccination efficacy rates increase. As can be seen from these visual findings, vaccination rates play a crucial role in controlling infections. The infected population decreases as the fractional order increases. Furthermore, simulations were conducted to demonstrate the dynamics of daily COVID-19 cases in the UAE when vaccination coverage increased by 50% and 100%. Figure 6 and Figure 9 illustrate the resulting graphical interpretation. The graphs in Figure 6 show that a higher vaccination rate significantly reduces the peaks of infected curves. In particular, a 100% increase in vaccination coverage can significantly reduce infection peaks and even eradicate the disease. Therefore, these results demonstrate that if the vaccination rate is high enough and the vaccines are used effectively, pandemics can be eliminated not only in the chosen region but also globally.

**Remark** **4.** *A mathematical model serves as a theoretical basis for formulating and simulating epidemic prevention measures, as well as a tool for predicting and analyzing epidemic spread. The fractional model has been found to be more effective than the integer-order model [36,58]. The purpose of this study is to investigate possible issues arising from vaccination for COVID-19. In [5], the authors examined whether multiple vaccination strategies could affect COVID-19 dynamics in a population using the Atangana-Baleanu derivative. Here, we present the dynamics of the time-delayed COVID-19 disease model using the Caputo fractional derivative.*

## 5. Conclusions

A three-dimensional time-delayed fractional-order COVID-19 mathematical model was investigated with vaccination efficacy. The COVID-19 vaccines were highly effective, but the epidemic was difficult to control despite their effectiveness. One of the reasons is that I(t) converges to a non-zero constant after bifurcation. Before becoming an endemic illness, COVID-19 coexisted with individuals for a substantial time period. The model has both disease-free and endemic equilibria, and it is locally asymptotically stable. If $\tau >\tau_{0}$, bifurcation appears in the vaccination model. To achieve thorough and rapid COVID-19 control, several non-pharmaceutical methods such as reducing the transmission rate and isolating more asymptomatic individuals must be appropriately implemented in addition to population vaccination. The model was examined by fitting it to real observations in the UAE for the time period from 25 March to 30 July 2020 for the population without vaccination, 15 July to 15 December 2021 for 50% of the vaccinated population, and 15 January to 20 May 2022 for 100% of the vaccinated population.

To choose the most effective approach for treatment, control, and elimination of the infection, control variables should be included in the model. A sensitivity analysis can also identify the essential parameters, which could serve as an important threshold in disease management. These will be taken into account in future research.

## Figures and Tables

**Table 1:** Descriptions of model (1)’s variables and parameters.

Parameter	Description	Value Range	References
δ	Transmission rate of symptomatic individuals	[0,1)	[48,49]
$V_{ac}$	Vaccination rate	0.01/day	Assume
ν	Vaccine efficacy rate	[0.8,0.9]	Assume
β	Symptomatic infectious disease recovery rate	[0,1)	[50]

**Table 2:** Reproduction number $R_{0}$ and bifurcating point $\tau_{0}$.

Transmission Rate δ	Recovery Rate β	Reproduction Number $R_{0}$	$\omega_{0}$	$\tau_{0}$
0.30	0.15	2.00	0.6269	3.8192
0.32	0.15	1.77	0.7430	3.7078
0.35	0.20	1.75	0.8290	3.6818
0.39	0.23	1.6956	0.9422	3.7368
0.42	0.25	1.6800	1.0237	3.8759
0.45	0.28	1.6071	1.1477	4.5782

**Table 3:** Fractional-order α and bifurcating point $\tau_{0}$.

Fractional-Order α	Critical Frequency $\omega_{0}$	Bifurcating Point $\tau_{0}$
0.810	1.6075	5.1136
0.815	1.5756	4.4790
0.820	1.3602	3.7445
0.825	1.2538	3.681
0.830	1.1472	3.6714
0.835	1.10864	3.6808

**Table 4:** Vaccination rate $V_{ac}$ and bifurcating point $\tau_{0}$.

Vaccination Rate $V_{\mathrm{ac}}$	Critical Frequency $\omega_{0}$	Bifurcating Point $\tau_{0}$
0.75%	1.000	6.108
0.78%	1.002	5.305
0.81%	1.002	4.862
0.84%	1.007	4.554
0.87%	1.009	4.317
0.90%	1.012	4.125

**Table 5:** Vaccine efficacy rate ν and bifurcating point $\tau_{0}$.

Vaccine Efficacy Rate ν	Critical Frequency $\omega_{0}$	Bifurcating Point $\tau_{0}$
90%	1.005	4.768
86%	1.002	5.272
82%	0.999	6.313
88%	1.004	4.990
84%	1.001	5.665
80%	0.998	8.642

## Data Availability

The data presented in this study are available on request from the corresponding author. The data are not publicly available due to privacy or ethical restrictions.
